# c-Jun N-terminal kinase signaling in aging

**DOI:** 10.3389/fnagi.2024.1453710

**Published:** 2024-08-29

**Authors:** Yihao Li, Li You, Eugenie Nepovimova, Vojtech Adam, Zbynek Heger, Klaudia Jomova, Marian Valko, Qinghua Wu, Kamil Kuca

**Affiliations:** ^1^College of Life Science, Yangtze University, Jingzhou, China; ^2^College of Physical Education and Health, Chongqing College of International Business and Economics, Chongqing, China; ^3^Department of Chemistry, Faculty of Science, University of Hradec Králové, Hradec Králové, Czechia; ^4^Department of Chemistry and Biochemistry, Mendel University in Brno, Brno, Czechia; ^5^Department of Chemistry, Faculty of Natural Sciences and Informatics, Constantine the Philosopher University in Nitra, Nitra, Slovakia; ^6^Faculty of Chemical and Food Technology, Slovak University of Technology, Bratislava, Slovakia; ^7^Andalusian Research Institute in Data Science and Computational Intelligence (DaSCI), University of Granada, Granada, Spain

**Keywords:** JNK, aging, molecular insights, therapeutic targets, longevity

## Abstract

Aging encompasses a wide array of detrimental effects that compromise physiological functions, elevate the risk of chronic diseases, and impair cognitive abilities. However, the precise underlying mechanisms, particularly the involvement of specific molecular regulatory proteins in the aging process, remain insufficiently understood. Emerging evidence indicates that c-Jun N-terminal kinase (JNK) serves as a potential regulator within the intricate molecular clock governing aging-related processes. JNK demonstrates the ability to diminish telomerase reverse transcriptase activity, elevate β-galactosidase activity, and induce telomere shortening, thereby contributing to immune system aging. Moreover, the circadian rhythm protein is implicated in JNK-mediated aging. Through this comprehensive review, we meticulously elucidate the intricate regulatory mechanisms orchestrated by JNK signaling in aging processes, offering unprecedented molecular insights with significant implications and highlighting potential therapeutic targets. We also explore the translational impact of targeting JNK signaling for interventions aimed at extending healthspan and promoting longevity.

## 1 Introduction

Aging encompasses a gradual process of physiological, biochemical, and psychological changes that occur in organisms over time, resulting in a progressive decline in their functional and adaptive capacity (Mitina et al., 2020; Xu et al., 2020). This decline affects multiple systems and organs, leading to a weakening of their functions (Madison and Kiecolt-Glaser, 2021; Pietri and Stefanadis, 2021; van Gastel and Carmeliet, 2021; Yousefzadeh et al., 2021). Aging also brings about a reduction in the quantity and quality of cells in the body, resulting in organ and tissue atrophy (Dave et al., 2022; Sasaki et al., 2022; Li H. et al., 2023; Li N. et al., 2023). Specifically, the circulatory, respiratory, and digestive systems are profoundly impacted by aging (Schuliga et al., 2021; Jiang and Zhao, 2022; Abdellatif et al., 2023). Furthermore, aging is associated with a decline in immune function, increasing susceptibility to infections and tumors (Kim K. M. et al., 2022; Girotra et al., 2023). Age-related changes in the nervous system can lead to cognitive decline and neurodegenerative diseases (Huang et al., 2021). Consequently, understanding the molecular mechanisms underlying aging is of utmost importance. The underlying mechanisms of aging are intricate and multifaceted. Over the past decade, various factors, including cellular senescence, telomere attrition, stem cell exhaustion, deregulated nutrient-sensing, and altered intercellular communication, have been recognized as playing critical roles in the aging process (Deng et al., 2021; Wiley and Campisi, 2021; Lopez-Otin et al., 2023; Topiwala et al., 2023). Notably, recent studies have unveiled the connection between circadian rhythms and aging (Acosta-Rodriguez et al., 2021; Hodge et al., 2022). Disruption of circadian rhythms heightens the risk of stem cell senescence and chronic diseases associated with aging, such as neurodegenerative diseases and osteoarthritis (Liang et al., 2022; Nassan and Videnovic, 2022; Zhu et al., 2022). Moreover, dysbiosis of the gut microbiota has been implicated in promoting aging (Mossad et al., 2022; Walters, 2023). The gut microbial community is a key regulator of immune homeostasis (Jensen et al., 2021). Age-related alterations in the gut microbiota contribute to the deterioration of immune system function, hasten the development of immunosenescence and inflammatory aging, impair brain function, and accelerate aging-related cognitive decline (Boehme et al., 2021; Bosco and Noti, 2021). These studies have significantly advanced our understanding of aging. However, the upstream regulatory pathways that govern these factors within the context of aging have not been fully explored yet.

In our previous work, we have discussed the function of c-Jun N-terminal (JNK) signaling in the regulation of cellular senescence [details can be read in Deng et al. (2023)]. Cellular senescence is considered to be a process of organism aging (Lopez-Otin et al., 2023). Moreover, a growing body of evidence indicates the involvement of JNK signaling in the organism aging process (Gan et al., 2021; Lee et al., 2022; Yin et al., 2022). Telomeres, known to undergo significant shortening after multiple rounds of cell division, contribute to cellular senescence and aging (Shoeb et al., 2021; Rossiello et al., 2022). JNK signaling can facilitate the reduction of the telomerase catalytic subunit hTERT, resulting in the shortening of telomere terminal restriction fragments (TRFs) and telomere depletion. This, in turn, contributes to the deceleration of the bone marrow microenvironment and immune system aging (Yoon et al., 2015; Abdul-Aziz et al., 2019). Additionally, JNK has the potential to modulate intercellular communication by downregulating MMP-1 mRNA expression and extracellular matrix (ECM) degradation in human dermal fibroblasts (nHDFs), thereby contributing to skin aging (Gao et al., 2018; Choi et al., 2022; Lopez-Otin et al., 2023). Importantly, JNK phosphorylation is enhanced in the absence of the circadian rhythm protein BMAL1, resulting in reduced β-catenin expression and GSK-3β phosphorylation. This attenuation of osteoblast differentiation and mineralization further contributes to skeletal aging (Li H. et al., 2023; Li N. et al., 2023; Novakovsky et al., 2023). Abnormal activation of JNK signaling promotes the proliferation and differentiation of intestinal stem cells (ISCs) through the upregulation of Wnt signaling. This upregulation subsequently increases the expression of biomarkers Ccdn1, Axin2, and Lgr5, leading to ISC exhaustion and intestinal tract aging (Jiang H. et al., 2016). Moreover, JNK also exhibits the capacity to modulate gut microbiota and promote the aging process. JNK signaling can be activated by lipopolysaccharide (LPS) stimulation, promoting hippocampal neuronal apoptosis and cognitive decline, thus contributing to brain aging (Jiang W. et al., 2016; Yang et al., 2023). Furthermore, dual oxidase (DUOX)/ROS activates JNK signaling in the presence of gut microbiota dysbiosis, accelerating brain aging by exacerbating the progression of Parkinson’s disease (Liu et al., 2022). Collectively, these studies highlight the crucial role played by JNK signaling in the regulation of the aging process. However, there are still research gaps and limitations in this field. The comprehensive study of JNK in aging mechanisms, particularly regarding intestinal homeostasis dysregulation and circadian rhythm disorders, remains incomplete. The precise mechanisms by which inflammatory factors regulate intestinal microbiota imbalance and circadian rhythm protein disorder through JNK signaling, leading to aging, are not fully understood. Additionally, the subtypes of JNK are often overlooked in these studies. Exploring the specificity of JNK, including its isoenzymes and substrates, in the regulation of the aging process is necessary for the development of anti-aging interventions. Furthermore, JNK-targeting drugs tend to focus more on the aging-promoting aspects of JNK while neglecting its dual role in the regulation of the aging process.

Hence, within this framework, we provide a comprehensive outline of recent research advances contributing to elucidating the molecular mechanisms underlying the role of JNK signaling in aging process. We delve into the therapeutic potential of targeting JNK in aging and outline the current challenges that persist in this field. Our particular emphasis lies in exploring the regulatory effects of JNK on telomere dysfunction, stem cell exhaustion, intercellular communication, circadian rhythm proteins, and gut microbiota to unravel the intricate mechanisms underlying aging. The elucidation of these molecular mechanisms underscores the significance of JNK as a pivotal molecular target in the context of aging.

## 2 JNK modulates telomere function and telomerase activity

**Figure 1:**
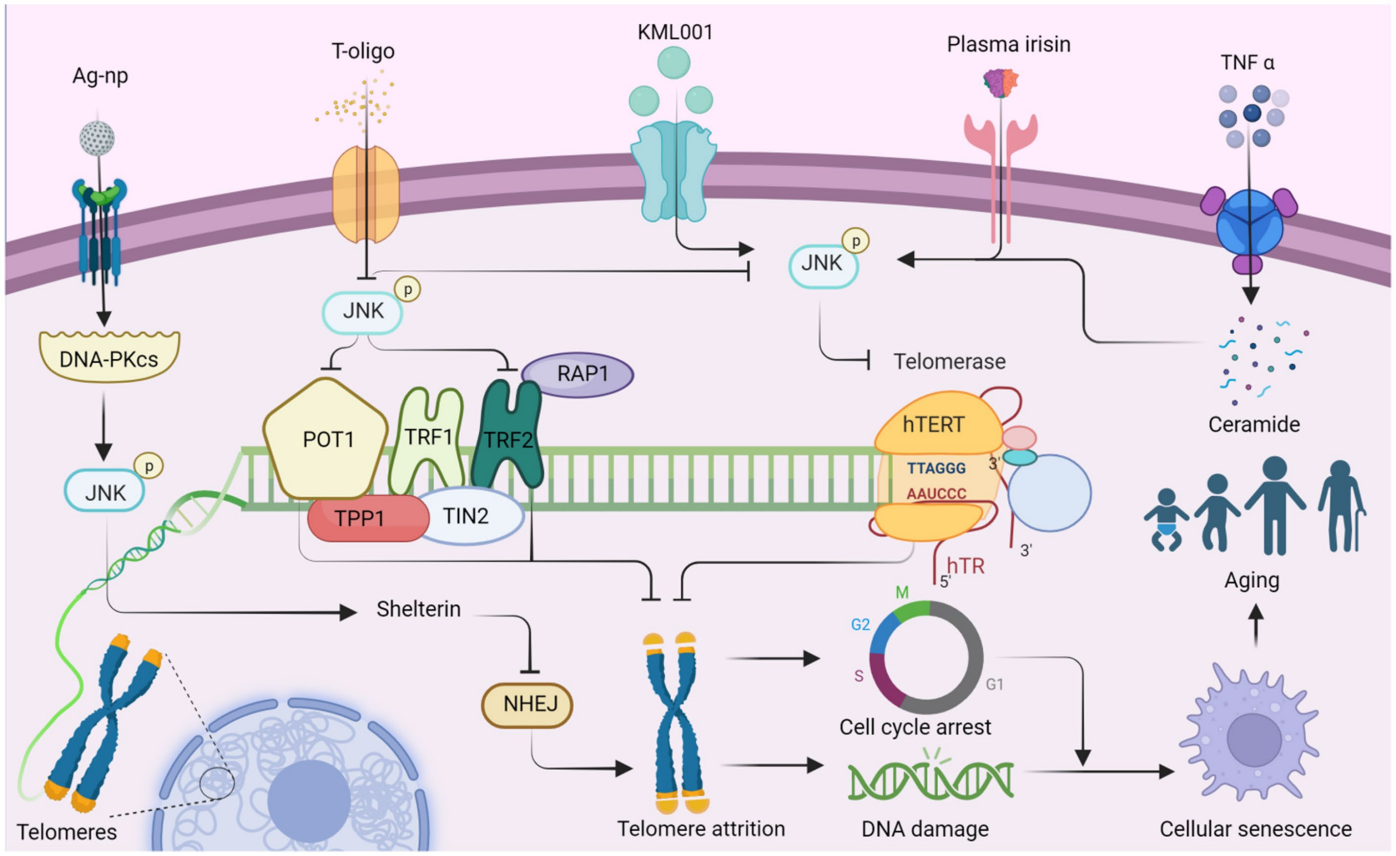
JNK affects telomere attrition by regulating the production of telomere-binding proteins and telomerase, which ultimately leads to organismal aging. Ag-NP stimulates the production of DNA-PKcs, promotes JNK phosphorylation, and stimulates the production of the telomere-binding protein complex shelterin. Inhibition of NHEJ (non-homologous end joining) attenuates telomere attrition. Additionally, T-oligo inhibits JNK activity and suppresses the production of telomere-binding proteins POT1 and TRF2, which in turn promotes telomere attrition. Conversely, T-oligo promotes telomerase production by inhibiting JNK phosphorylation, ultimately inhibiting telomere depletion, whereas KML001 has the opposite effect. Both elevated plasma irisin levels and TNF-α-promoted ceramide production increase JNK phosphorylation, which subsequently negatively regulates hTERT production and accelerates telomere attrition. Ultimately, telomere attrition can contribute to cell cycle arrest and DNA damage, leading to cellular senescence and individual aging. This figure is drawn by the authors using Biorender.

Telomeres, the protective caps of chromosomes consisting of nuclear proteins and DNA, play a crucial role in maintaining genome stability (Denham, 2020). These specialized structures, rich in guanine, are particularly vulnerable to oxidative damage (Prasad et al., 2017). Telomeres naturally shorten with each cell division and as a consequence of aging, and their damage is closely associated with the aging process and age-related diseases (Rossiello et al., 2022; Dasanayaka et al., 2023). Furthermore, the expression and activity of telomerase are involved in the regulation of telomere length (Hoffmann et al., 2021). Substantial evidence suggests that JNK signaling can modulate the expression of telomere-binding proteins, as well as telomerase expression and activity, thereby influencing the aging process (Chhabra et al., 2018; Bi et al., 2020) (Figure 1).

Telomerase, a key enzyme responsible for maintaining telomere length and stability, consists of several factors and accessory proteins, including hTERT and hTR, which preserve telomere length through reverse transcriptase activity (Deng et al., 2019). TNF-α stimulation induces sphingomyelin hydrolysis, resulting in ceramide production and subsequent activation of JNK in leukemic KG1 cells. This activation leads to a decrease in hTERT activity, causing prolonged growth arrest, increased β-galactosidase activity, telomere shortening, and severe chromosomal instability (Beyne-Rauzy et al., 2005). Therefore, JNK activation may plays a role in reducing hTERT activity and promoting telomere shortening. In a similar way, JNK inhibitors have shown potential as therapeutic agents for leukemia. For instance, sodium metaarsenate (NaAs2O3: KML001), an orally available arsenic compound, up-regulating phosphorylation of PTEN, ERK, p38, and JNK (Yoon et al., 2015). KML001 treatment results in a decrease in the catalytic subunit of telomerase (hTERT) and shortening of the telomere terminal restriction fragment (TRF), leading to G1 arrest and apoptosis in *HL-60* and *HL-60R* cells (Yoon et al., 2015). KML001 may regulate telomere shortening and apoptosis through the MAPK pathway, which is intricately linked to the aging process. Under ischemia–reperfusion conditions, elevated plasma irisin levels inhibit JNK phosphorylation, enhance hepatocyte hTERT activity, and lengthen telomeres, ultimately increasing the level of repair in senescent liver (Bi et al., 2020).

T-oligo, a guanine-rich oligonucleotide that mimics the 3′-telomeric overhang of telomeres, exhibits antiproliferative activity (Wojdyla et al., 2014). Inhibition of JNK partially reverses the antiproliferative effects of T-oligo and reduces the mRNA expression of telomerase reverse transcriptase. Conversely, JNK inhibitors reverse the expression of the catalytic subunit of telomerase, promoting DNA damage and apoptosis (Chhabra et al., 2018). T-oligo may up-regulate POT1/TRF2 and that JNK activation inhibits human telomerase reverse transcriptase (Chhabra et al., 2018). JNK may promote proliferation within melanoma cells and be beneficial to the cells themselves. Shelterin, a six-subunit protein complex, binds to human telomere ends, safeguarding chromosome ends from DNA damage and maintaining telomere length (Erdel et al., 2017). Silver nanoparticles (Ag-np) are toxic to the entire genome of eukaryotic cells, triggering DNA-PKcs (DNA-dependent protein kinase, catalytic subunit) and activating JNK1 and NHEJ, which repairs the telomere-located shelterin complex (Lim et al., 2017). This finding demonstrates a favorable aspect of Ag-np activation of JNK signaling during the process of cell senescence. The interplay between the subunits of Shelterin and the CTC1-STN1-TEN1 (CST) complex and their relationship with JNK remains to be studied (Gavia-Garcia et al., 2021).

JNK plays a crucial role in regulating telomere maintenance and the aging process by modulating telomerase activity and the function of telomere-binding proteins. The length and stability of telomeres are closely associated with longevity. Exciting advancements have been made in the field of telomere lengthening. For instance, T cells acquire telomeres from antigen-presenting cells (APCs), resulting in increased telomere length and a more youthful appearance in the T cells themselves. Furthermore, extracellular vesicles containing telomeres obtained from mouse and human blood have successfully extended telomere length in human T cells, outperforming the activity of telomerase by 30 times (Lanna et al., 2022). In the future, it may be possible to transfer telomeres from the blood cells of other animals to the human body. However, it is important to consider targeted delivery methods to ensure that telomere extension is beneficial and selective, especially when dealing with harmful cells such as cancer cells.

## 3 JNK governs stem cells exhaustion in aging

JNK signaling not only affects telomere function and telomerase activity but also plays a role in regulating stem cell exhaustion during the aging process (Bi et al., 2020; Zhang et al., 2021). Stem cell exhaustion or dysfunction is a significant indicator of aging (Yousefzadeh et al., 2021; Lopez-Otin et al., 2023). Stem cells possess the ability to self-renew and differentiate into various cell types, which is crucial for injury and disease repair, tissue regeneration, and maintaining homeostasis (Fu et al., 2021). However, as individuals age, the number of stem cells gradually decreases, and their functionality declines, leading to a loss of tissue integrity and deteriorating health (Hegab et al., 2019; Faust et al., 2020). Abnormal activation of JNK signaling can also impact the differentiation ability and functional expression of stem cells, resulting in their inability to effectively carry out repair and regeneration tasks (Xiao et al., 2019; Yu et al., 2019). This further affects tissue and organ function, contributing to the aging process at the organismal level.

Adult hematopoietic stem cells (HESCs) gradually lose their proliferative capacity after undergoing multiple divisions. However, when KMT2C-deficient hematopoietic stem cells are cultured with IL-1, they retain their multilineage potential and fail to activate JNK and p38. Thus, the deletion of the KMT2C gene may protect HESCs from exhaustion and holds potential for the treatment of acute myelogenous leukemia (AML) (Chen et al., 2019). Additionally, the dysfunction of wild-type HESCs can potentially be addressed using JNK inhibitors. For example, the JNK signaling pathway inhibitor JNK-IN-8, when used to culture human cord blood (CB) CD34(+) cells, enhances the self-renewal of HESCs (Xiao et al., 2019). Similarly, knockdown of c-Jun, a major downstream target of JNK, promotes the expansion of hematopoietic stem progenitor cells (HSPCs) (Xiao et al., 2019). These findings collectively demonstrate the role of JNK signaling in the renewal and exhaustion of HESCs.

Heat shock protein 70 (HSP70) is a family of cytoprotective and antioxidant proteins (Elmallah et al., 2020). In various contexts, HSP70 reduces ROS production, downregulate the JNK/c-Jun pathway, restore mitochondrial membrane potential (MMP), and prevent ATP depletion. These protective effects of HSP70 contribute to the preservation of the ultrastructure of nucleus pulposus stem cells (NPSCs), prevention of neural stem cell exhaustion, and preservation of the number and quality of neural stem cells (Zhang et al., 2021). Furthermore, mitochondrial fusion plays a crucial role in maintaining pluripotency during somatic cell reprogramming and induced pluripotent cell (iPSC) generation. For instance, sustained mitochondrial fusion accompanies the differentiation of *Drosophila* intestinal stem cells. In contrast, intestinal stem cells with inhibited mitochondrial fusion display lower mitochondrial membrane potentials, reduced ATP levels, elevated ROS levels, and impaired differentiation capacity. Inhibition of fusion also hinders progenitor cell differentiation, while scavenging ROS partially rescues the differentiation defects associated with mitochondrial fusion deficiency by downregulating JNK activity (Deng et al., 2018). Moreover, during neural stem cell (NSC) differentiation, decreased Wnt/β-linker activity and increased Wnt/AP-1 activity are observed. Silencing of the ATF2 gene inhibits JNK activity and NSC differentiation (Bengoa-Vergniory et al., 2014). Additionally, the JNK-involved extracellular matrix (ECM) signaling pathway can influence stem cell differentiation. For example, HMGB1 released by reactive astrocytes stimulates NSC differentiation to facilitate brain injury repair via the HMGB1/RAGE/JNK pathway (Li et al., 2014).

In an aging *Drosophila* model, the ingestion of caffeic acid inhibits the oxidative activation of JNK signaling, reduce aberrant proliferation, and extending the lifespan of senescence-associated intestinal stem cells. Additionally, supplementation of Ca^2+^ restores intestinal defects caused by oxidative stress downstream of JNK signaling (Yousefzadeh et al., 2021). The aberrant activation of JNK in intestinal stem cells may promote senescence in Drosophila. It would be valuable to investigate whether calcium supplementation or JNK suppression could also be beneficial for combating aging and increasing longevity in humans. Furthermore, it remains unclear whether Ca^2+^ interacts directly with intestinal stem cells or with the intestinal flora. Wnt signaling is a crucial pathway involved in the proliferation of intestinal stem cells, and the JNK-c-Jun/AP-1 pathway upregulates the expression of Wnt genes. This, in turn, upregulates the expression of Ccdn1, Axin2, and Lgr5, promoting the proliferation and differentiation of intestinal stem cells (Jiang H. et al., 2016).

**Figure 2:**
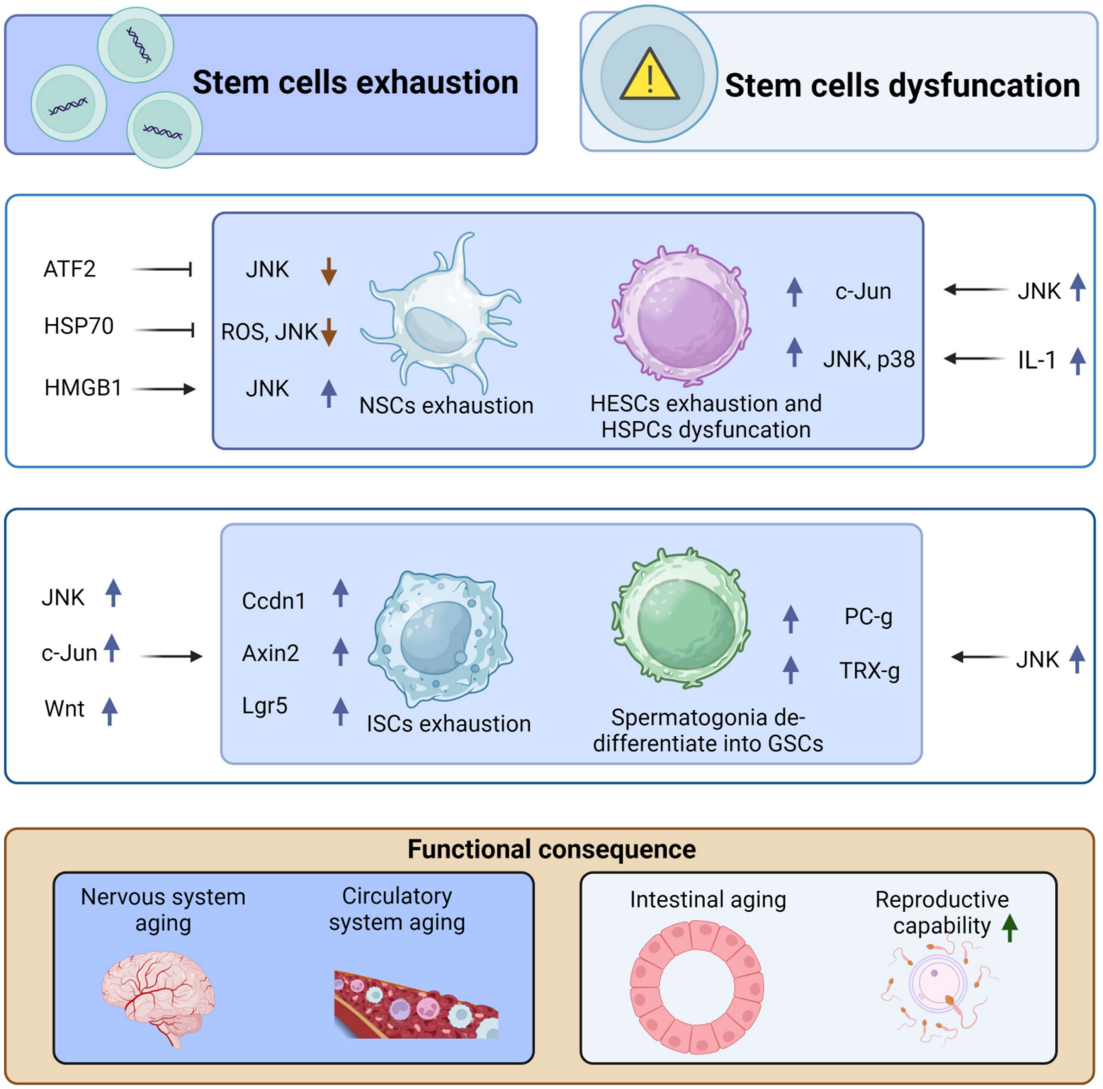
JNK regulates organismal aging by mediating stem cell exhaustion and dysfunction. High-mobility group box 1 (HMGB1) can stimulate the differentiation of neural stem cells (NSCs), repair brain damage, and inhibit aging by upregulating JNK. However, both activating transcription factor-2 (ATF2) and HSP70 can inhibit the differentiation and exhaustion of NSCs by suppressing JNK activation. Similarly, IL-1 stimulates the upregulation of p38 and JNK, promoting the exhaustion of adult hematopoietic stem cells (HSCs) and, in turn, causing aging of the circulatory system. Moreover, JNK can cause dysfunction of hematopoietic stem progenitor cells (HSPCs) through the upregulation of c-Jun, which further contributes to aging. In addition, JNK induces the proliferation and differentiation of intestinal stem cells (ISCs) through the upregulation of c-Jun and Wnt signaling, which in turn upregulates the expression of Cyclin D1 (Ccnd1), Axin2, and Lgr5, ultimately leading to intestinal stem cell exhaustion and aging. However, JNK stimulates dedifferentiation to form germline stem cells (GSCs) through the upregulation of Polycomb-group (Pc-g) proteins or trithorax-group (trx-g) genes, thus avoiding exhaustion of GSCs and delaying organismal aging. This figure is drawn by the authors using Biorender.

In the testis of male *Drosophila*, the number of germline stem cells (GSCs) decreases with age. JNK signaling triggers the de-differentiation of spermatogonia during chronic stress to maintain a pool of GSCs in the *Drosophila* testis. Polycomb-group (PC-g) or trithorax-group (TRX-g) genes downstream of JNK may confer proliferative capacity to dedifferentiated GSCs, resulting in increased proliferative capacity (Herrera and Bach, 2018). The genetic map may undergo alterations that need to be confirmed through further experiments, and the signaling pathway is not yet fully understood. JNK plays a significant role in stem cell dysfunction and is generally detrimental. However, its behavior differs in intestinal stem cells (ISCs) and neural stem cells (NSCs), where it exhibits favorable effects. Additionally, there is a complex interplay between JNK signaling and embryonic stem cells and cancer stem cells (Latham et al., 2022; Novakovsky et al., 2023). Hypoactivation of JNK signaling in intestinal progenitor cells leads to apoptosis and exhaustion of ISCs, and high levels of expression of the senescence marker DCP-1, which affects intestinal regeneration and promotes intestinal aging (Zhang et al., 2024). In summary, the mechanisms by which JNK signaling regulates aging through stem cell failure or dysfunction involve changes in the proliferation and renewal capacity of stem cells (Figure 2).

## 4 JNK alters intercellular communication during the aging process

**Figure 3:**
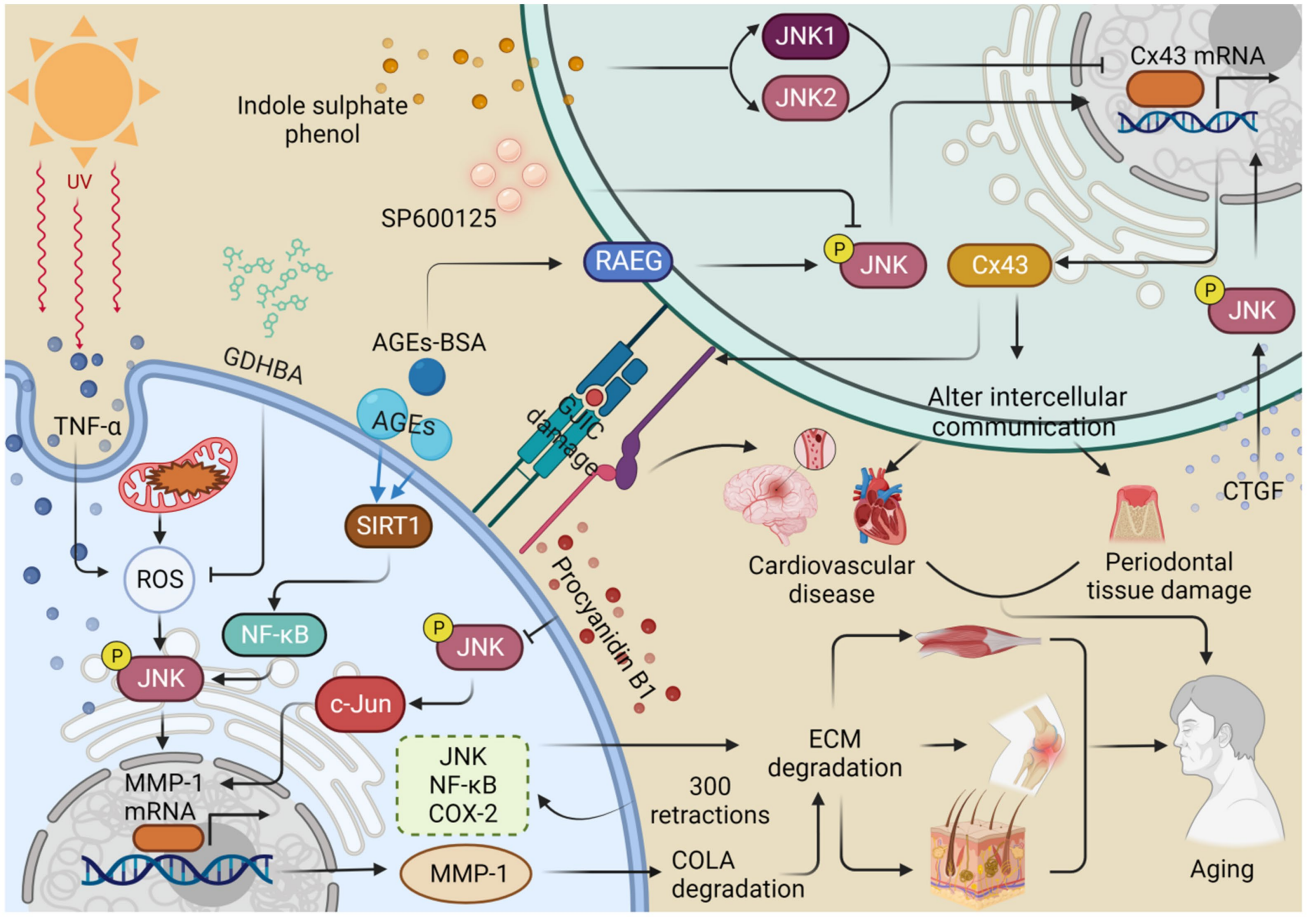
JNK regulates the expression of MMP-1 and Cx43, which alters intercellular communication and thus contributes to organismal aging. Ultraviolet A (UVA) stimulates the production of TNF-α and reactive oxygen species (ROS), promotes the phosphorylation of JNK, and enhances the expression of MMP-1. This process contributes to the degradation of collagen (COL1A) and the extracellular matrix (ECM), consequently accelerating skin aging. 2-O-beta-D-glucopyranosyl-luteolin (GL) and procyanidin B1 inhibit MMP-1 production by inhibiting ROS production and JNK phosphorylation, respectively, thus retarding collagen degradation and skin aging. Advanced glycation end products (AGEs) promote JNK phosphorylation and MMP-1 expression via the SIRT1/NF-κB pathway, resulting in collagen degradation, ECM deposition, and eventually osteoarthritis. Repetitive stretching, performed 300 times, increased the expression of the JNK/NF-κB/COX-2 pathway, resulting in abnormalities in the ECM and muscle aging. Indole sulfate (IS) stimulates the production of JNK1 and JNK2 and inhibits the expression of Cx43, leading to a disruption of gap junction intercellular communication (GJIC) and ultimately contributing to cardiovascular senescence. The inhibitor SP600125 reduces JNK phosphorylation and decreases the expression of Cx43, which can result in damage to periodontal tissue. In contrast, connective tissue growth factor (CTGF) improves intercellular communication by promoting JNK phosphorylation to upregulate Cx43 expression. Similarly, treatment with AGEs-breakers (AGEs-BAS) leads to overexpression of the receptor for advanced glycation end products (RAGE) and JNK phosphorylation, which promotes Cx43 expression and delays the aging process. This figure is drawn by the authors using Biorender.

Stem cell exhaustion is intimately associated with disrupted intercellular communication, and restoring such communication holds the potential to counteract stem cells exhaustion (Mas-Bargues et al., 2020). The JNK pathway plays a pivotal role in the aging process and age-related diseases, which are influenced by the dysregulation of intercellular communication (Yan et al., 2018). Intercellular communication can be established through direct cell-to-cell contact or regulated by cytokines over long distances (Fafian-Labora and O'Loghlen, 2020). Altered intercellular communication represents a hallmark of senescence, with senescent cells actively engaging in cellular communication with each other and neighboring cells through various modes of communication (Fafian-Labora and O'Loghlen, 2020; Lopez-Otin et al., 2023). Perturbed intercellular communication primarily arises from changes in connexin (Cx) levels, extracellular matrix (ECM) remodeling, and JNK’s involvement in these pathways is instrumental in regulating the aging process (Yan et al., 2018; Choi et al., 2022) (Figure 3).

### 4.1 JNK influences the expression of the messenger molecular connexin43

Connexins (Cxs) form gap junctions that facilitate the exchange of inorganic ions and messenger molecules. Among the various connexin isoforms, connexin43 (Cx43) is the most critical and widely distributed (Xu Q. et al., 2021; Cao et al., 2023). Impaired intercellular coupling and reduced Cx43 levels in gap junctions have been associated with increased JNK activation in intact atrial cells and with aging. Chronically activated JNK suppresses the transcriptional activity of the Cx43 gene promoter through c-Jun, resulting in downregulated Cx43 expression. This impairment of intercellular communication contributes to the development of atrial fibrillation (AF) (Yan et al., 2018). Additionally, upon stimulation with indole sulphate phenol (IS), JNK1/2 phosphorylation is further elevated in rat cardiomyocytes and *H9c2* cells, leading to decreased Cx43 protein and mRNA levels. Inhibition of p-JNK attenuates the IS-induced decrease in Cx43 transcription and translation, as well as the disruption of gap junction intercellular communication (GJIC) between cardiomyocytes, ultimately contributing to cardiovascular disease (Changchihen et al., 2020). Thus, inhibiting JNK may be an effective strategy for preventing AF and cardiovascular diseases in the elderly. Moreover, Cx43 plays a crucial role in maintaining the functional integrity of the vascular endothelium. Treatment with advanced glycation end products (AGEs)-BAS results in the overexpression of the receptor for advanced glycation end products (RAGE) and the activation of JNK expression, ultimately leading to a significant increase in Cx43 protein levels in human umbilical vein endothelial cells (HUVECs). However, the JNK inhibitor SP600125 can reverse this increase in Cx43 expression (Zhao et al., 2019). Interestingly, JNK also plays a protective role in the vascular endothelium, highlighting the dual nature of JNK’s effects. Thus, long-term JNK activation may have detrimental consequences.

The interplay between the Cx43 and JNK pathways is implicated in periodontitis, which accelerates aging and increases mortality (Wang et al., 2023). During severe stress, Cx43-mediated gap junctions exacerbate tissue damage. However, both 18-a-glycyrrhetinic acid (GA) and SP600125 inhibit Cx43 expression and reduce oxidative stress and apoptosis through the JNK/NF-κB pathway, thereby attenuating oxidative damage in periodontal tissues (Cao et al., 2023). Intercellular communication is crucial for human periodontal ligament stem cells (hPDLSCs) to respond to biological stimuli and maintain microenvironmental homeostasis. Among hPDLSCs, Cx43 and Panx1 are the most widely expressed gap junction hemichannels. Connective tissue growth factor (CTGF) induces Akt, JNK, and p38 phosphorylation, promoting subcellular migration and upregulating Cx43 and Panx1 to enhance intercellular communication. Notably, Panx1 expression is primarily dependent on PI3K/Akt signaling (Wu et al., 2022a). Therefore, JNK can modulate Cx43 levels to regulate senescence, and intriguingly, JNK can both promote and repress Cx43 expression. Thus, when using JNK inhibitors for regulation, targeted delivery is necessary to precisely transport them to the intended lesion and avoid damage to healthy tissues (Figure 3).

### 4.2 JNK contributes to the remodeling of the extracellular matrix

The extracellular matrix (ECM) is a dynamic and organized network of proteins and carbohydrates that is synthesized and secreted by cells (Chakraborty and Edkins, 2021). Disruptions in ECM homeostasis can contribute to various diseases. For instance, ECM hardening is the underlying cause of cartilage sclerosis and knee joint inflammation, while ECM degradation is associated with skin aging (Lee et al., 2022; Iijima et al., 2023). Disturbed ECM homeostasis can also lead to increased intraocular pressure (Agarwal and Agarwal, 2018). It is likely that JNK is involved in these processes. In AGE-induced SW1353 cells, treatment resulted in elevated levels of p-p65 and p65, p-JNK and JNK, and p-38 and p38, indicating that AGE can induce inflammation through the SIRT1/NF-kB/MAPK pathway, as well as the degradation of COL2A and aggrecan. These processes ultimately lead to inflammation, ECM deposition, and the development of osteoarthritis (OA) (Zhou et al., 2022). Although a SIRT1 inhibitor reversed this effect, the experiment did not include a JNK inhibitor, suggesting that a JNK inhibitor might have a similar effect.

The dermis of human skin contains an ECM composed of collagen, elastin, and other proteins, which decrease with aging (Yousefzadeh et al., 2021). COLA in the ECM is cleaved by matrix metalloproteinases (MMPs), and reactive oxygen species (ROS) also contribute to ECM degradation, thereby promoting skin aging (Choi et al., 2022). In human dermal fibroblasts (nHDFs) exposed to procyanidin B1, inhibition of p-JNK was observed, which suppressed the JNK/c-Jun axis and oxidative stress-mediated induction of MMP-1 mRNA expression, thus mitigating ECM degradation (Cha and Kim, 2015). UV radiation induces the production of the pro-inflammatory cytokine TNF-α, which stimulates the production of MAPKs, nuclear NF-κB, and AP-1, leading to the expression of collagenase MMP-1. Treatment with 2-O-beta-D-glucopyranosyl-14,6-dihydroxybenzaldehyde (GDHBA) from mulberry fruits effectively inhibited ROS production, reduced NO and PGE2 levels, decreased the expression of p38, ERK, JNK, c-Jun, NF-κB, and cyclooxygenase-2 (COX-2), and ultimately decreased MMP-1 secretion, increased COL1A secretion, and inhibited ECM degradation (Kim K. S. et al., 2022). Proanthocyanidin B1, found in natural ingredients, may serve as a potential JNK inhibitor with anti-photoaging properties. These natural and organic ingredients hold great potential for both health and skincare products. In hairless rats exposed to Ultraviolet A (UVA) radiation, increased ROS production, JNK phosphorylation, MMP-1 production, and degradation of COL1A affected the ECM and disrupted intercellular communication, ultimately contributing to skin aging (Lan et al., 2019).

ECM abnormalities have been associated with skeletal muscle aging. After 300 muscle elongation contractions, aged muscles exhibited unchanged levels of glycoproteins Tenascin C and fibronectin, increased inflammatory responses, elevated and delayed expression of monocyte chemotactic protein-1 (MCP-1), increased expression of NF-κB/p38/JNK, and abnormalities in ECM and MAPK responses compared to younger muscles (Sorensen et al., 2018). These findings highlight the role of JNK inhibition in regulating ECM homeostasis and maintaining overall organismal health (Figure 3).

## 5 JNK signaling in cellular senescence

Intercellular communication and cellular senescence are closely linked, with extracellular vesicles playing a crucial role in facilitating this communication and contributing to cellular senescence and age-related diseases (Yin et al., 2021). Extensive evidence supports the involvement of the JNK pathway in cellular senescence (Deng et al., 2023). Cellular senescence is characterized by cell cycle arrest, enlarged morphology, telomere damage, inflammatory response, endoplasmic reticulum stress, mitochondrial dysfunction leading to excessive ROS production, DNA methylation, and histone modifications (Kamal et al., 2020; Lopez-Otin et al., 2023). Senescent cells secrete a range of factors known as the senescence-associated secretory phenotype (SASP), which is a hallmark of cellular senescence (Xu et al., 2014). Although cellular senescence itself does not directly cause aging, the SASP factors secreted by senescent cells can propagate senescent phenotypes to neighboring cells (Admasu et al., 2023). When the accumulation of senescent cells reaches a certain level, SASP factors become prominent, ultimately leading to organ failure, disease, and death (Ovadya et al., 2018). In other words, the degeneration of bodily functions is caused by the buildup of senescent cells. Removing senescent cells inhibits the progression of related diseases. For example, the removal of senescent cardiomyocytes and non-cardiomyocytes in aged female rats promotes cardiac remodeling and regeneration after myocardial infarction (Salerno et al., 2022). Quercetin effectively eliminates senescent bone marrow mesenchymal stem cells in the elderly, promoting the repair of bone defects (Xing et al., 2022). The JNK, p38-MAPK pathway, GMP-AMP synthase (cGAS)-stimulator of interferon genes (STING) pathway, and NOTCH pathway all contribute to the production of SASP factors (Vizioli et al., 2020). The accumulation of senescent cells in various tissues contributes to aging and age-related diseases, such as Alzheimer’s disease, cardiovascular sclerosis, and diabetes complications, which are strongly associated with cellular senescence (Hoffman et al., 2016; Reddy et al., 2017; Abdul-Aziz et al., 2019; Palmer et al., 2021).

Immune cell senescence is particularly concerning as it accelerates the senescence of other cells, leading to overall aging of the body (Deng et al., 2024). Immune cells undergo age-related functional alterations. Sestrin forms the immunosuppressive sestrin MAPK activation complex (SMAC) by binding to mitogen-activated protein kinases (MAPKs), promoting the activation of extracellular signal-regulated kinase (ERK), JNK, and p38. SMAC is more likely to form in T cells of individuals over 65 years old, and its elimination restores antigen-specific functional responses (Lanna et al., 2017). The reduction of naive T cells and B cells is an important marker of immune aging. In the elderly, TNF-α induces apoptosis of naive T cells and central memory T cells, and the expression of TRAF-2 and RIP, phosphorylation of JNK, IKKα/β, and IκBα, as well as the activation of NF-κB, are significantly reduced in both types of T cells, indicating a link between reduced JNK activation, T cell senescence, and immune aging (Gupta et al., 2018). Treatment of macrophages with ACR induces increased senescence-associated β-galactosidase (SA-β-gal) activity and G0/G1 phase arrest. Knockdown of Activating transcription factor 3 (ATF3) eliminates all ACR-induced senescence symptoms (Kim et al., 2015). ATF3 activates p38 and JNK kinases, which promote ATF3-dependent p53 expression, leading to macrophage senescence (Kim et al., 2015). However, most studies have focused on the cellular inflammatory response and apoptosis in activated T cells, and the relationship between immune cell senescence and the JNK pathway remains to be investigated. Interestingly, high TNFR2 expression leads to JNK overexpression, which blocks the polarization and differentiation of senescent MDSCs, suggesting that JNK activation may lead to a reduction in the differentiation of MDSCs into mature granulocytes, dendritic cells (DCs), and macrophages, further contributing to immune aging (Wang et al., 2023).

**Figure 4:**
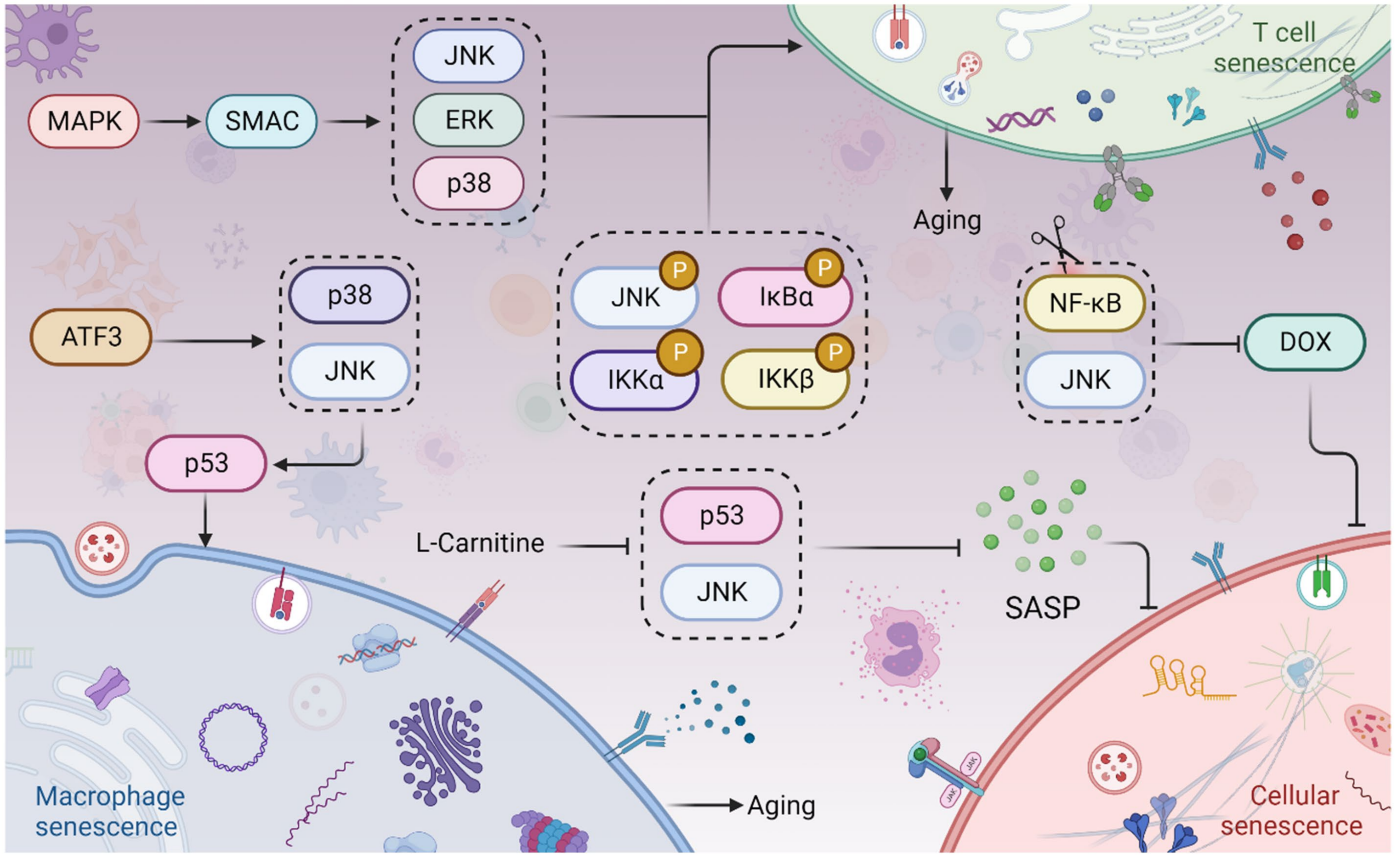
JNK signaling in cellular senescence and aging. The Sestrin MAPK Activation Complex (SMAC) binds to mitogen-activated protein kinases (MAPKs), facilitating the activation of extracellular signal-regulated kinase (ERK), JNK, and p38. This leads to T cell senescence and aging. Phosphorylation of JNK, IKKα/β, and IκBα also contributes to T cell senescence and aging. Moreover, Activating Transcription Factor 3 (ATF3) induces the activation of p38 and JNK kinases, which in turn promotes p53 expression, culminating in macrophage senescence. Interestingly, inhibition of the JNK and NF-κB pathways can prevent doxorubicin (Dox)-induced cellular senescence. Furthermore, L-Carnitine has been shown to attenuate the Senescence-Associated Secretory Phenotype (SASP) by inhibiting the JNK/p53 pathway. This figure is drawn by the authors using Biorender.

JNK plays a role in cell cycle arrest, which subsequently affects cellular senescence. The signaling of JNK influences the expression of m6A methylase FTO, leading to endothelial cell senescence. This is evidenced by an increase in positive staining for senescence-associated β-galactosidase and a higher proportion of cells arrested in the G0/G1 phase (Li H. et al., 2023; Li N. et al., 2023). Doxorubicin (Dox) is a drug used for cancer treatment that induces senescence and enhances the secretion of SASP factors in endothelial cells (ECs). On the other hand, metformin is a commonly used drug for diabetes treatment (Pampurik et al., 2019; Du et al., 2023). Metformin counteracts the upregulation of senescence markers induced by Dox and reduces the secretion of SASP factors and adhesion molecules. It significantly inhibits the JNK and NF-κB pathways and can be clinically utilized to prevent Dox-induced vascular aging during anticancer therapy (Abdelgawad et al., 2023) (Figure 4). The degeneration of intervertebral discs (IDD) involves the aging and programmed cell death of nucleus pulposus cells (NPCs) (Jia et al., 2023). Kaempferol treatment partially reverses the IL-18-induced reductions in levels of aggregated proteoglycan, COL2A, SOX9, and FN1. Moreover, it significantly attenuates the IL-18-stimulated promotion of p38, JNK, and ERK1/2 phosphorylation. Kaempferol treatment also improves the IL-18-induced inhibition of NPCs’ viability and senescence (Wang et al., 2023).

Adipocyte hypertrophy is associated with cellular senescence, collagen deposition, and systemic inflammation (Ye R. et al., 2022). Adipose tissue is widely distributed throughout the body and serves as a significant source of chronic sterile inflammation (Stout et al., 2017). With aging, age-related changes occur in the composition of adipose tissue. Adipose tissue inflammation pathologically leads to adipocyte hypertrophy and fibrosis (Li H. et al., 2023; Li N. et al., 2023). In human adipose tissue, increased JNK phosphorylation in senescent macrophages contributes to inflammation. L-Carnitine attenuates adipose tissue SASP in the elderly by inhibiting the JNK/p53 pathway, thereby reducing chronic inflammation in aging adipose tissue (Yang et al., 2019). This attenuation of senile adipose tissue dysfunction and improvement in insulin resistance suggest possible functional similarities between L-Carnitine and JNK inhibitors.

## 6 JNK, circadian rhythm, and aging

Senescent cells undergo functional decline, contributing to various detrimental effects, including impaired circadian oscillations that play a role in the development of age-related diseases (Cha et al., 2022; Hashikawa et al., 2023). There is a close correlation between circadian rhythm protein expression and JNK phosphorylation (Uchida et al., 2012; Yoshitane et al., 2012). The aging process is closely intertwined with circadian rhythms, which exhibit decreased amplitude with age (Sato et al., 2017; Schibler, 2020). Circadian clock genes express circadian rhythm proteins that regulate biological aging, metabolic processes, cancer, and age-related degenerative diseases (Musiek and Holtzman, 2016; Pan et al., 2020; Feng et al., 2022). Notable circadian rhythm proteins include brain and muscle arnt-like protein 1 (BMAL1), circadian locomotor output cycles kaput (CLOCK), Period (PER) 1–2, Cryptochrome (CRY) 1–2, and Timeless (TIM) (Zhao et al., 2018; Zhang et al., 2023). Furthermore, mutations in circadian rhythm proteins genes have been associated with the development of chronic diseases and the aging process in humans, highlighting the crucial role of circadian rhythm proteins in maintaining healthy tissues and resisting aging (Sochal et al., 2023). Down-regulation, absence, or denaturation of circadian rhythm proteins can impair the functioning of biological clocks, subsequently affecting sleep quality, immune function, and metabolic regulation, ultimately contributing to a range of age-related diseases (Shin and Lee, 2021; Sochal et al., 2023).

BMAL1 is a prominent circadian rhythm protein, and its deficiency is linked to age-related diseases, including blindness (Dubrovsky et al., 2010; Chhunchha et al., 2020). Osteoporosis is a condition that primarily affects the elderly due to age-related decline in bone mass, which is closely associated with osteoblast (OB) function (Kim et al., 2021; Li H. et al., 2023; Li N. et al., 2023). Interestingly, BMAL1 expression and JNK phosphorylation are involved in this process. Knockdown of BMAL1 in osteoblasts leads to increased phosphorylation of ERK and JNK, enhanced mTOR activity, reduced expression of β-catenin, and decreased phosphorylation of GSK-3β (Wei et al., 2023). Therefore, the circadian gene BMAL1 may regulate osteoblast differentiation and inflammatory responses in a JNK/ERK/mTOR/GSK3β/β-catenin-dependent manner, resulting in impaired osteoblast differentiation and mineralization (Li H. et al., 2023; Li N. et al., 2023; Novakovsky et al., 2023). Additionally, calcific aortic valve disease (CAVD), which is common in the elderly, is associated with BMAL1 and calcification (Gomez-Stallons, 2016; Li H. et al., 2023; Li N. et al., 2023). BMAL1 expression is elevated in calcified human aortic valves and valve interstitial cells (VICs) derived from calcified human aortic valves (Jiang et al., 2023). Knockdown of BMAL1 inhibits the osteogenic differentiation of VICs by reducing the levels of p-JNK, leading to suppressed osteogenic differentiation (Li H. et al., 2023; Li N. et al., 2023). Furthermore, BMAL1 deficiency is also associated with skin aging (Li et al., 2022). Aging skin becomes dry, dehydrated, develops wrinkles, loses its radiance, and experiences hair graying and loss (Souto et al., 2020; Amini et al., 2022). Photoaging caused by ultraviolet A (UVA) radiation is a major factor in skin aging induced by solar radiation (Tsuchida et al., 2023). There is a direct correlation between liver metabolism and JNK activation, neutrophil elastase (NE) levels, and BMAL1 expression. NE inhibits JNK, which in turn inhibits FGF21 and activates the expression of the BMAL1 gene in hepatocytes. Neutrophils and their elastase are involved in a pathological condition associated with reduced hepatic lipogenesis, suggesting that neutrophils contribute to maintaining daily liver homeostasis by regulating the NE/JNK/BMAL1 axis, thus supporting daily metabolic homeostasis (Crespo et al., 2020). In gastrointestinal syndromes, the absence of BMAL1, a core circadian rhythm protein, disrupts the circadian clock and rhythmic proliferation. BMAL1 can stimulate the JNK stress response via TNF, promoting the proliferation of intestinal epithelial precursor cells in a pathological state (Stokes et al., 2017).

CRY is a crucial circadian protein in mammals, and CRY1/2 have distinct roles in regulating circadian rhythms (Parico and Partch, 2020). Originating from DNA repair enzymes, CRY proteins safeguard genomic integrity through coordinated transcriptional regulation (Papp et al., 2015). Defects in CRY1 affect the length and stability of the circadian cycle, while defects in a single allele of CRY2 impact circadian rhythms (Oda et al., 2022). Knockdown of CRY2, another type of circadian rhythm protein, decreases p53 expression, increases cyclin D1 expression, and enhances p-ERK but not p-JNK, suggesting that CRY2 does not directly regulate aging or age-related diseases by modulating JNK activity. Deletion of the CRY2 gene significantly increases mRNA expression of CRY1, PER1/2, BMAL1, and CLOCK (Yu et al., 2018), indicating the involvement of CRY in the aging process. Currently, there are no studies exploring interaction of CRY1 and JNK in aging.

In mouse glial cells, knockdown of the PER1 gene leads to increased β42 deposition and expression of misfolded proteins during senescence, suggesting an association between PER1 and neurodegenerative diseases (Boerner et al., 2021). Norepinephrine (NA) can further regulate PER1 mRNA expression through the PKA/JNK-CREB and ERK/JNK-c-Jun pathways in rat C6 cells, implying the involvement of JNK in PER1 expression, which in turn affects aging (Sugimoto et al., 2011). Peripheral thyroid clock genes, particularly BMAL1, PER mRNA, and PER2 protein, are down-regulated in the thyroid gland of aged mice. The dysregulation of the circadian rhythm of PER2 during aging activates the AP-1 transcription factor via the JNK pathway, potentially contributing to age-related thyroid hyperplasia (Lee et al., 2022). TIM, another important CRP found in various species (such as Drosophila, mice, and humans), regulates the transcriptional loops of core circadian rhythm genes and is involved in aging (Li et al., 2016; Top et al., 2016; Kotwica-Rolinska et al., 2022). However, the connection between JNK and aging through TIM has not been studied yet. Interestingly, overexpression of TIM delays cellular senescence (Shen et al., 2018). TIM is highly expressed in several cancers due to circadian deregulation, but there are no studies directly linking it to JNK and aging. Regarding CLOCK proteins, several studies have demonstrated their association with aging and age-related diseases (Neilsen et al., 2019; Bi et al., 2020; Yoshida et al., 2021). For instance, mutations in the CLOCK gene are associated with conditions such as shortened lifespan, cardiovascular disease, obesity, and metabolic syndrome (Dubrovsky et al., 2010; Shin and Lee, 2021; Soni et al., 2021; Shon et al., 2022).

**Figure 5:**
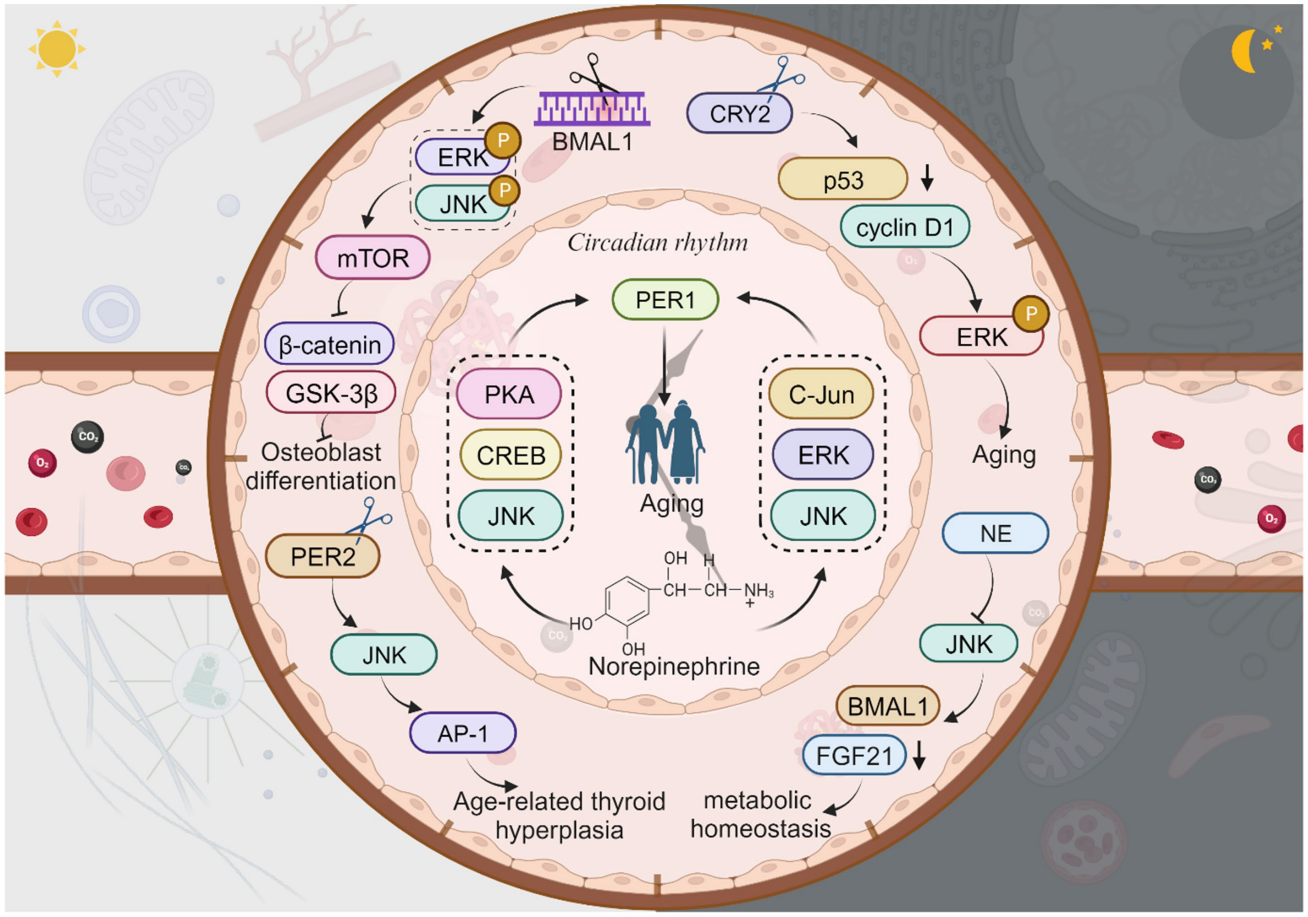
The JNK signaling with circadian rhythm proteins and aging. Knockdown of BMAL1 leads to increased phosphorylation of ERK and JNK, enhanced mTOR activity, reduced expression of β-catenin, and decreased phosphorylation of GSK-3β, resulting in impaired osteoblast differentiation. Neutrophil elastase (NE) inhibits JNK, which in turn inhibits FGF21 and activates the expression of the BMAL1 gene, thus supporting daily metabolic homeostasis. Knockdown of CRY2 decreases p53 expression and increases cyclin D1 expression, then enhances p-ERK, thus directly regulate aging or age-related diseases. Norepinephrine regulates PER1 mRNA expression through the PKA/JNK-CREB and ERK/JNK-c-Jun pathways which in turn affects aging. The dysregulation of PER2 activates the AP-1 transcription via the JNK pathway, contributing to age-related thyroid hyperplasia. This figure is drawn by the authors using Biorender.

Extensive research is currently underway to investigate the intricate relationship between circadian rhythm proteins such as BMAL1, PER1-3, CRY1-2, TIM, and CLOCK, and the process of aging. Disruptions in circadian rhythms and abnormalities in these circadian rhythm proteins have been implicated in hastening the onset of aging. Consequently, maintaining a robust circadian rhythm is crucial for attenuating the aging process. Circadian rhythm proteins exhibit a dual role in aging, capable of either delaying or promoting the aging process. Notably, the absence of BMAL1 is associated with lens degeneration and neurodegenerative diseases (Chhunchha et al., 2020). However, the majority of studies have predominantly focused on the impact of JNK in diminishing the expression of circadian rhythm proteins such as BMAL1 and PER in diseases associated with aging. While the roles and interrelationships among circadian rhythm protein molecules have been extensively explored, limited research has been conducted to elucidate the connection between JNK and TIM or CRY (Figure 5).

## 7 The influence of JNK on intestinal dysbiosis and its role in the aging process

**Figure 6:**
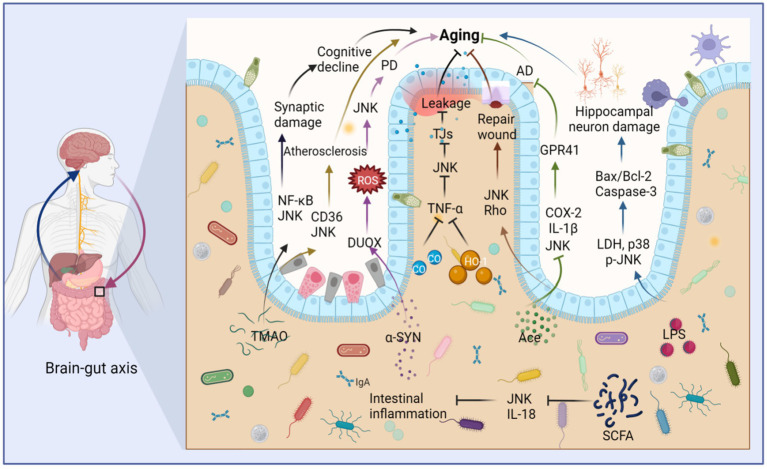
Intestinal microbiota metabolites stimulate JNK to regulate intestinal barrier destruction and aging through the brain-gut axis. Trimethylamine oxide (TMAO) stimulates the upregulation of JNK and NF-κB, which cause synaptic damage and aging. TMAO also stimulates the upregulation of JNK and CD36, leading to atherosclerosis and aging. Furthermore, alpha-synuclein (α-SYN) contributes to Parkinson’s disease (PD) and aging by promoting the production of DUOX and stimulating the upregulation of ROS and JNK. LPS induces the expression of lactate dehydrogenase (LDH), p38, and JNK, thereby promoting the production of Bax/Bcl-2 and causing aging of hippocampal neurons. In contrast, acetate (Ace) can repair intestinal epithelial damage by stimulating the upregulation of JNK and Rho. Ace also suppresses the expression of JNK, IL-1β, and COX-2, thus fostering the production of G-protein-coupled receptor 41 (GPR41), ultimately inhibiting Alzheimer’s disease (AD) and aging. Both carbon monoxide (CO) and heme oxygenase-1 (HO-1) limit the production of TNF-α, thereby inhibiting JNK activity, reducing the destruction of tight junctions (TJs), and mitigating damage to the intestinal barrier, thus inhibiting aging. Short-chain fatty acids (SCFAs) can inhibit JNK activation and IL-18 production, suppressing intestinal inflammation and aging. This figure is drawn by the authors using Biorender.

The circadian clock plays a crucial role not only in regulating the aging process but also in orchestrating the 24-h oscillation of gastrointestinal epithelial structure and function (Fawad et al., 2022). Furthermore, circadian proteins have been found to significantly influence microbiota dynamics, and the transfer of microbiota from mice with impaired circadian clock genes has been shown to induce altered gut phenotype (Heddes et al., 2022). Interestingly, JNK has also been implicated in the regulation of aging by disrupting intestinal homeostasis (Li et al., 2020) (Figure 6). The human intestine harbors a diverse array of bacteria that contribute to food digestion, absorption, immune regulation, and various other physiological activities (Seidel and Valenzano, 2018; Victorelli et al., 2023). The microbiota of the intestine, often referred to as the “second human gene pool,” forms a complex symbiotic relationship with the human body (Xu Y. et al., 2021). Remarkably, the gut microbiota and the brain engage in bidirectional communication through the brain-gut-axis, playing crucial roles in normal physiological processes and the pathogenesis of numerous human diseases (Chen et al., 2021). It becomes evident that human lifespan and aging are closely intertwined with gut flora and its metabolites, and modulating gut flora can potentially delay the aging process (Du et al., 2021). During aging, notable changes occur in the composition and function of gut microorganisms, including a decline in probiotic bacteria (Conway and Duggal, 2021).

### 7.1 Intestinal flora metabolites coordinately regulate aging mediated by JNK

Age-related dysregulation of the intestinal ecosystem exacerbates inflammatory factors and the production of harmful substances, leading to impaired intestinal barrier function. Consequently, various deleterious metabolites can infiltrate the body through the intestines, triggering aging processes and age-related diseases (Usui et al., 2018). For instance, trimethylamine (TMA), which is extensively metabolized by gut flora, undergoes hepatic oxidation catalyzed by liver enzymes to form trimethylamine oxide (TMAO) (Dalla Via et al., 2020). TMAO activates inflammatory signaling pathways such as NF-κB, NOD-, LRR-, and pyridine-containing domain protein 3 (NLRP3) inflammasomes, as well as MAPK/JNK in peripheral tissues and the brain (Praveenraj et al., 2022). Elevated TMAO levels are associated with age-related cognitive dysfunction and can induce mitochondrial dysfunction, neuronal aging, and synaptic damage in the brain (Praveenraj et al., 2022). Additionally, TMAO induction may induce foam cell formation and contribute to atherosclerosis through the CD36/MAPK/JNK pathway (Geng et al., 2018).

Nevertheless, it is important to note that not all intestinal metabolites have detrimental effects. Bile acids (BAs), for example, play an essential role in the brain-gut axis, promoting intestinal cell regeneration and activating intestinal stem cells (Sorrentino et al., 2020). However, metabolic function slows down with age, and gut dysbiosis can lead to abnormalities in the gut metabolome (Milenkovic et al., 2022). BAs are also implicated in metabolism, with miR-26a playing a key role in glucose regulation and lipid metabolism. Activation of the TGR5 receptor requires the JNK pathway for miR-26a induction (Chen et al., 2015). Furthermore, certain intestinal strains can produce lipopolysaccharide (LPS), which, when stimulated, results in increased lactate dehydrogenase (LDH) release, elevated levels of phosphorylated JNK and p38, an increased Bax/Bcl-2 ratio, enhanced Caspase-3 expression, and ultimately promotes neuronal apoptosis in the hippocampus (Cai et al., 2017).

Short-chain fatty acids (SCFAs) are generated through the bacterial fermentation of dietary fiber and play a crucial role in maintaining intestinal homeostasis. They impact intestinal motility, enhance the integrity of the intestinal barrier, and influence host metabolism (Martin-Gallausiaux et al., 2021). However, dysbiosis can lead to a reduction in SCFA-producing bacteria (Igarashi et al., 2017). Among the neurodegenerative diseases prevalent in the elderly, Alzheimer’s disease (AD) stands out. Acetate (Ace), a neuroprotective SCFA, has shown promising effects in this context. Oral Ace administration in mice inhibited the phosphorylation of NF-κB p65, ERK, and JNK. It also decreased the levels of COX-2 and IL-1β in Aβ-stimulated BV2 microglia and elevated G-protein coupled receptor 41 (GPR41) levels in BV2 cells, ultimately restoring cognitive function (Ding et al., 2020). Furthermore, Ace has demonstrated its potential as a therapeutic agent for intestinal mucosal injury by promoting wound healing in mouse colonic epithelial cells through the activation of JNK and Rho signaling pathways (Nakano et al., 2018). In a TNF-α-induced *Caco-2* cell model, SCFAs and phenolic metabolites exhibited synergistic anti-inflammatory effects by downregulating the gene expression of IL-8, TNF-α, and VCAM-1. They also inhibited the phosphorylation of JNK, p38, and IκBα, affecting cellular signaling mediators of the MAPK and NF-κB pathways (Zheng et al., 2021). Although the mechanisms through which SCFAs exert their effects *in vivo* are complex, limited studies have explored their relationship with JNK. However, it is worth noting that the aforementioned metabolites may regulate the JNK pathway during intestinal aging, although the specific molecular mechanisms remain unknown (Figure 6).

### 7.2 JNK regulates the brain-gut axis to promote aging process

The brain-gut axis is a complex and interactive system comprising neural, endocrine, immune, and intestinal flora components. It plays a crucial role in maintaining human health and regulating age-related diseases (Kelly et al., 2015). Changes in the gut microbiota, such as an increase in *Bifidobacterium* spp., *Clostridium* spp., *Aspergillus*, and *Enterobacteriaceae,* have been associated with aging (Jeffery et al., 2016). Interestingly, centenarians possess an abundant microbial ecosystem consisting of *Bifidobacterium*, *Akkermansia*, *and Christensenellaceae*, which has been hypothesized to be linked to longevity (Kong et al., 2016). Within the brain-gut axis, there exist two natural signaling barriers: the intestinal barrier and the blood–brain barrier (BBB) (Martin et al., 2018). During the aging process, these barriers may be compromised, resulting in increased BBB permeability, slowed intestinal motility (Montagne et al., 2015; Pentinmikko and Katajisto, 2020), and reduced barrier integrity. Consequently, conditioned pathogens and toxic metabolites can translocate into the body, triggering systemic inflammation, a key factor in many age-related diseases (Wang J. et al., 2020; Wu et al., 2022b). Aging also leads to structural changes in intestinal epithelial cells, resulting in a decline in barrier function. The apoptotic and renewal capacity of these cells diminishes with age, further contributing to the decline in barrier function. Additionally, the aging gut experiences decreased levels of IgA, TLR4, CD4+, and CD8+, leading to reduced T-cell signaling pathways and promoting chronic low-grade inflammation (Sovran et al., 2019). As age progresses, the abundance of *A. mucilaginosa* decreases, contributing to increased gut permeability and the circulation of pro-inflammatory factors (Bodogai et al., 2018). Furthermore, studies comparing young and aging mice have revealed detrimental alterations in gut microbiota, reduced cerebral blood flow, impaired cognitive function, and heightened inflammatory markers in aging mice (Hoffman et al., 2016). These findings suggest a significant association between aging and the brain-gut axis.

Although research on the interplay between the brain-gut axis, JNK, and aging remains relatively limited, emerging evidence highlights the pivotal role of JNK signaling in driving the progression of neurodegenerative diseases through the brain-gut axis in *Drosophila*, as well as its involvement in disrupting the integrity of the intestinal epithelial barrier in mice (Figure 6). Parkinson’s disease (PD) has been closely associated with JNK activation and dysbiosis of the intestinal flora (Liu et al., 2022). Notably, the aberrant aggregation of alpha-synuclein (α-SYN) stands as a key pathological hallmark of PD (Ryu et al., 2019). In a *Drosophila* gut model, the presence of intestinal α-SYN triggers the generation of reactive oxygen species (ROS), propelling the acceleration of PD progression via the dual oxidase (DUOX)-ROS-JNK pathway. This cascade culminates in a compromised lifespan, loss of dopaminergic neurons, and progressive motor deficits (Liu et al., 2022). Furthermore, age-related metabolic imbalances are exacerbated by the disruption of the intestinal epithelial barrier (Tatge et al., 2023). Moreover, in murine models, the intricate involvement of the JNK signaling pathway in regulating the disruption of the intestinal barrier has been unveiled. For instance, heme oxygenase-1 (HO-1) and carbon monoxide (CO) cleave caspase-3 expression by effectively inhibiting the TNF-α-induced surge in the disruption of epithelial tight junctions (TJs). Simultaneously, they exert inhibitory effects on the phosphorylation of ERK, p38, and JNK, thus safeguarding the integrity of the intestinal barrier by fortifying intestinal TJs and mitigating apoptosis in intestinal epithelial cells (Zhou et al., 2021). These findings underscore the intricate interplay between the TNF-α-activated MAPK pathway, heightened JNK phosphorylation, TJs disruption, apoptosis of intestinal epithelial cells, and the consequential disruption of the intestinal barrier. Nevertheless, it is worth noting that the overexpression of HO-1 exacerbates age-related heart failure (Allwood et al., 2014). JNK has also emerged as a key player in intestinal inflammation, and inflammatory bowel disease (IBD) has been closely associated with dysbiosis of the intestinal microbiota, intestinal aging, and a decline in the abundance of the mucin-producing bacterium Akkermansia. In an experimental model of Dextran sodium sulfate (DSS)-induced IBD, the activation of ER stress markers EIF2a and JNK within intestinal epithelial cells was observed. Interestingly, the presence of mucin Akkermansia counteracts the expression of TJs-2 in the leaky epithelium, effectively impeding the formation of paracellular leaky water channels. Consequently, this ameliorated intestinal inflammation and relieved epithelial cell stress associated with the maintenance of intestinal integrity (Wade and Su, 2021).

The JNK signaling pathway plays a significant role in neurodegenerative disorders and the disruption of the intestinal epithelial barrier through its regulation of brain-gut axis interactions. However, there is a dearth of comprehensive research elucidating the complete pathway of JNK signaling and its association with aging-related disorders. The pathway is involved in the progression of inflammation by influencing the integrity of the intestinal barrier, encompassing processes such as epithelial apoptosis, cell adhesion, and ligand proteins. Activation of the JNK pathway triggers an imbalance in the intestinal flora, characterized by an increase in pathogenic microorganisms and a decrease in beneficial bacteria. This imbalance promotes intestinal inflammation, oxidative stress, and hampers the repair of the intestinal mucosa, all of which have been linked to aging. Additionally, the JNK signaling pathway can impact the transport of microbial metabolites from the intestine to the liver, thereby exerting effects on liver function. Dysregulation of the intestinal flora leads to the release of metabolites such as LPS and amino acid metabolites, which in turn influence the host’s immune system and metabolic function, resulting in inflammatory responses and oxidative stress. These biological changes can activate the JNK signaling pathway and promote aging-related molecular pathways involving p38, ERK, TGR, and others. To conclude, JNK directly influences the aging process by modulating the balance of the intestinal flora. Potential strategies for treating diseases and extending lifespan include supplementation with appropriate probiotics, fecal transplants, and improving the structure of the gut microbiota. Both interventions targeting the intestinal microecology and JNK inhibitors hold promise as future therapeutic approaches for aging-related diseases stemming from dysbiosis of the intestinal flora (Figure 6).

## 8 Role of JNK in pyroptosis mediated aging

Disruption of gut homeostasis can trigger the production and release of inflammatory factors, activating the JNK signaling pathway. This activation, in turn, induces cellular pyroptosis characterized by the expression of CASP (caspase) and gasdermin (GSDM) proteins, while also impairing the integrity of the gut barrier (Chen et al., 2022). Additionally, this imbalance can trigger pyroptosis through the brain-gut axis, further exacerbating the aging process in organisms (Liao et al., 2023). Pyroptosis, also known as cellular inflammatory necrosis, enhances cell membrane permeability and mediates the release of inflammatory factors (Al Mamun et al., 2020). It is a regulated form of programmed cell death activated by inflammasomes and the gasdermin (GSDM) protein family, exerting its effects at the cellular level during aging (Liang et al., 2022). The GSDM family includes GSDMA, GSDMB, GSDMC, GSDMD, GSDME (also known as DFNA5), and DFNB59 (Ouyang et al., 2023). Following cellular pyroptosis and rupture of the cell membrane, inflammatory factors are released, affecting surrounding cells and triggering a local inflammatory response (Xie et al., 2021). Chronic inflammation is associated with advancing age, representing a characteristic feature of aging (Lopez-Otin et al., 2023). However, there is no clear evidence to suggest that cellular pyroptosis directly contributes to aging. Instead, it plays a role in diseases associated with aging, such as atherosclerosis, neurodegenerative diseases, hearing loss, and muscle degeneration, through cellular pyroptosis (Deng et al., 2021; Orberg et al., 2021; Zhao et al., 2021; Liang et al., 2022). These findings indicate the involvement of pyroptosis in the aging process.

**Figure 7:**
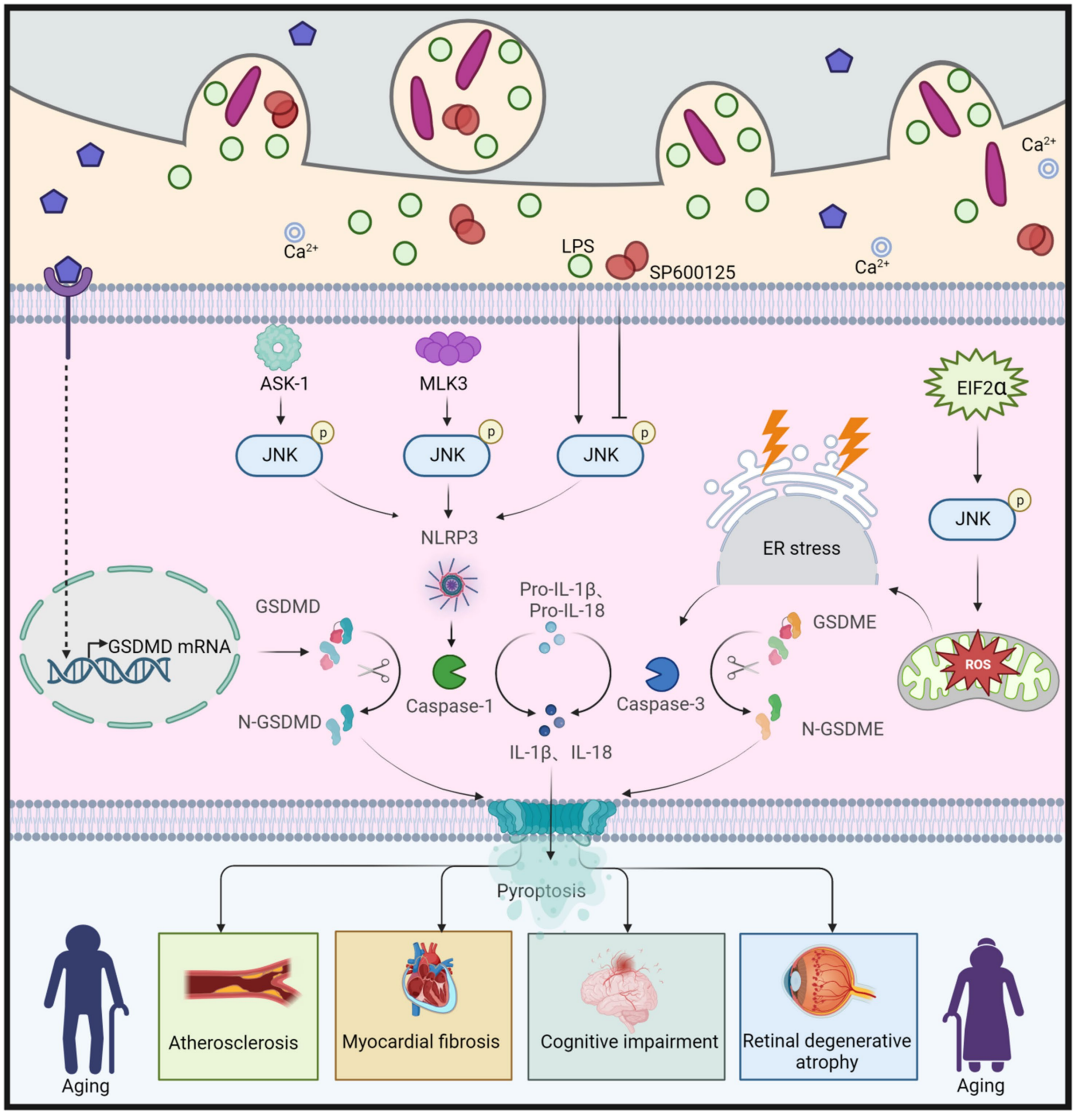
JNK regulation of GSDMD and GSDME leads to cellular pyroptosis, which in turn causes aging. Apoptosis signal-regulated kinase 1 (ASK-1) promotes the activation of JNK, which further converts proIL-1β, proIL-18, and GSDMD to IL-1β, IL-18, and N-GSDMD via the NLRP3 pathway, leading to cellular pyroptosis, and thus to atherosclerosis. Similarly, both Mixed Lineage Kinase 3 (MLK3) and LPS can cause cellular pyroptosis by enhancing JNK phosphorylation and promoting N-GSDMD production, leading to myocardial fibrosis and cognitive dysfunction, respectively. However, the JNK inhibitor SP600125 has been shown to slow down cellular pyroptosis by inhibiting JNK phosphorylation, which in turn attenuated cognitive dysfunction. Eukaryotic translation initiation factor 2α (EIF2α) promotes ROS production, enhances JNK phosphorylation, induces ER stress, and further increases caspase-3 expression, leading to increased production of N-GSDME, which promotes cellular pyroptosis and ultimately leads to retinal degeneration and atrophy. This figure is drawn by the authors using Biorender.

JNK is involved in the activation of different gasdermin (GSDM) proteins through multiple pathways, inducing various forms of pyroptosis and contributing to the aging process (Figure 7). The JNK pathway is likely positioned upstream of the NOD-like receptor protein 3 (NLRP3) inflammasome. Apoptosis signal-regulating kinase 1 (ASK-1) activates the JNK and NLRP3 pathways, leading to the conversion of proIL-1β, proIL-18, and GSDMD into IL-1β, IL-18, and N-GSDMD, respectively. Ultimately, this results in the deposition of thermoproteins and pyroptosis, contributing to atherosclerosis (Hoseini et al., 2018). Similarly, stimulation of NLRP3 and GSDMD in the presence of Cathepsin B induces cellular pyroptosis and promotes aging (Chang et al., 2017). Moreover, in the early stages of chronic heart failure (CHF), NF-κB/NLRP3-induced pyroptosis leads to myocardial fibrosis, while mixed lineage kinase 3 (MLK3) mediates inflammation (Wang J. et al., 2020). However, the involvement of JNK as an upstream signal of NF-κB is a topic of debate and requires further investigation. Additionally, immunoreactivity for NLRP3, GSDMD, and IL-1β is activated in BV2 microglia stimulated with lipopolysaccharide (LPS) (He et al., 2021). Conversely, the JNK inhibitor SP600125 inhibits the inflammatory response in BV2 cells, reducing the immune response to NLRP3 and GSDMD, thereby attenuating the progression of cognitive impairment (He et al., 2021). Furthermore, JNK is activated by eukaryotic translation initiation factor 2α (EIF2α), generating reactive oxygen species (ROS) and disrupting endoplasmic reticulum (ER) homeostasis, resulting in DNA damage, CASP3 expression, and promoting GSDME-mediated pyroptosis. Collectively, these mechanisms contribute synergistically to photoreceptor cell loss and retinal degenerative atrophy (Topiwala et al., 2023).

Indeed, JNK-mediated pyroptosis involving GSDMD and GSDME has been implicated in aging-related diseases, while the roles of other GSDM proteins remain poorly understood, and their mechanisms are unclear. Although direct evidence linking pyroptosis to aging is lacking, studies have indicated that pyroptosis may influence the onset and progression of aging through various mechanisms, including inflammatory responses, immune regulation, and oxidative stress. The anti-inflammatory effects of JNK inhibitors show promise in treating aging-related diseases associated with pyroptosis. However, the innate immune response is complex, and cellular pyroptosis is a highly intricate form of cell death regulated by multiple factors. Therefore, each potential therapeutic strategy must undergo rigorous validation through multiple studies.

## 9 Role of JNK in aging-related tissue/organ fibrosis

Pyroptosis, besides generating a significant amount of inflammatory factors, exerts diverse effects on the surrounding tissues (Wang et al., 2022). Notably, these factors play a pivotal role in the progression of tissue or organ fibrosis, primarily mediated by the JNK sigaling (Wang J. et al., 2020). Tissue or organ fibrosis is a common degenerative event that occurs during human aging and is associated with aging-related diseases such as hepatosclerosis, pulmonary fibrosis, renal fibrosis, and cardiac fibrosis (Mohammed et al., 2021; Yousefzadeh et al., 2021; Liang et al., 2022; Girotra et al., 2023). The JNK signaling pathway not only contributes to fibrosis in various tissues and organs but also represents a potential therapeutic target for pharmacological intervention in fibrotic diseases (Li et al., 2022).

In chronic liver disease, persistent hepatocellular injury and the resulting inflammatory response trigger the activation of hepatic stellate cells (HSCs) and promote the deposition of extracellular matrix (ECM), leading to hepatic fibrosis, which can progress to cirrhosis. There is a correlation between liver aging and the development of fibrosis (Qu et al., 2022). Fibroblast growth factor (FGF) 21 is closely associated with the JNK pathway and liver fibrosis. As liver fibrosis advances, the mRNA level of FGF21 gradually increases, and Interleukin (IL)-1β, through the NF-κB and JNK pathways, gradually enhances the expression of FGF21, thereby inhibiting liver fibrosis (Lee et al., 2018). Tumor necrosis factor superfamily 14 (TNFSF14), highly expressed in the context of fibrosis, binds to the lymphotoxin β receptor (LTβR) and activates JNK signaling, thereby regulating TGF-β1 expression, which can contribute to liver fibrosis *in vitro* (Liang et al., 2021). Bile acids (BAs) can function as signaling molecules, and a common receptor for them is the G protein-coupled bile acid receptor-5 (TGR5) (Zhang et al., 2022). Taurodeoxycholate (TDCA) and glycodeoxycholate (GDCA), mediated by TGR5, can activate HSC expression, stimulate JNK and ERK1/2 pathways, and increase the expression of NF-κB, IL-6, and TNF-α, thereby further promoting liver fibrosis (Xie et al., 2021).

During the induction of lung fibroblast apoptosis, a significant increase in p-JNK (phosphorylated JNK) was observed, indicating that JNK activation promotes the activation of lung fibroblasts. Idiopathic pulmonary fibrosis (IPF) is a severe and fatal lung disease that becomes more prevalent and severe with age. The use of anti-aging drugs has shown effectiveness in alleviating clinical symptoms in IPF patients (Wang J. et al., 2020), suggesting a connection between IPF and aging. FGF19 reduces apoptosis in alveolar type 2 epithelial cells by decreasing the expression of the pro-apoptotic BIM protein. Additionally, FGF19 prevents TGF-β-induced myofibroblast differentiation by inhibiting JNK phosphorylation. Therefore, FGF19 may possess resistance against pulmonary fibrosis and hold potential therapeutic implications for IPF (Justet et al., 2022). In a phase I study, CC-930, a JNK inhibitor, demonstrated a reduction in c-Jun phosphorylation induced by UV radiation in the skin. In preclinical models, CC-930 attenuated COL1A gene expression, indicating the involvement of JNK activity in the process of pulmonary fibrosis (van der Velden et al., 2016).

Activation of JNK induces cardiac fibrosis, and this pathological process is considered irreversible (Zhou et al., 2022). Under hypoxic conditions, HL-1 cells promote atrial fibrosis through the ROS/JNK pathway, leading to increased expression of fibrosis-related proteins such as COL1A and COL3A, TGF-β1, and α-SMA via downstream activation of nuclear c-Jun/ATF2 phosphorylation (Tsai et al., 2021). However, miR-let-7a, miR-let-7e, and miR-133a target the expression of COL1A and COL3A in HL-1 cardiomyocytes under hypoxia, attenuating hypoxia-induced cardiac fibrosis through post-transcriptional repression of the JNK pathway (Lo et al., 2022). Additionally, angiotensin II (Ang-II) promotes increased DNA content, COL1A and COL3A expression, and JNK activation in rat fibroblasts, whereas quercetin inhibits cardiac fibrosis by suppressing JNK phosphorylation and COLA expression through ROS inhibition (Zhang et al., 2019). Thus, JNK activation may contribute to COLA accumulation and cardiac fibrosis.

Renal fibrosis, which is age-related and contributes to the decline in renal function during aging, is a hallmark of chronic kidney disease (Chung et al., 2020; Wang Y. et al., 2020). MAPK phosphorylation, which can be negatively regulated by the dual specificity phosphatase (Dusp) subfamily, plays a role in the dephosphorylation and inactivation of phosphorylated JNK (Relav et al., 2021). Both Dusp1 and Dusp4 suppress the progression of renal fibrosis by inhibiting JNK activity (Laurent et al., 2019; Loeffler, 2019). Mitochondrial fission factor (MFF) induces mitochondrial fission, but Dusp1 overexpression inhibits JNK activity and can block or reduce the extent of mitochondrial damage. Similarly, Dusp4 inhibits JNK phosphorylation to slow down renal fibrosis, indicating that the JNK signaling pathway promotes renal fibrosis (Denhez et al., 2019; Sheng et al., 2019). The combination of Astragaloside IV (AS-IV) and ferulic acid (FA) also inhibits renal fibrosis by inhibiting JNK phosphorylation and reducing α-SMA expression (Meng et al., 2011).

In summary, JNK activation plays a significant role in various age-related fibrotic diseases. Current research indicates that there are distinct differences in JNK signaling pathways involved in organismal fibrosis, leading to both promotion and inhibition of renal fibrosis. While some studies have been conducted in mice, further investigation is needed to understand the mechanisms of JNK signaling in human tissue fibrosis. Additionally, fibrosis is a complex process involving multiple signaling pathways, including JNK, NF-κB, TGF-β, and potentially non-classical Wnt signaling. The interplay between these pathways requires further exploration. For a comprehensive overview of the effects and mechanisms of JNK signaling in aging (Table 1).

**Table 1:** Role of JNK signaling in aging process.

Target	Model	Treatment	Effect	Mechanism	Reference
Telomere attrition	Human *KG1* cells	TNF-α	Sphingomyelin hydrolysis, ceramide production and JNK are activated, β-gal activity is increased, and hTERT activity is decreased	JNK activation by TNF-α inhibits hTERT activity, resulting in telomere shortening, chromosome instability and aging	Beyne-Rauzy et al. (2005)
	*HL-60* and *HL-60R* cells	KML001	Strengthen PTEN, ERK, p38, and JNK phosphorylation, reduction of hTERT, and shortening of TRF length led to G1 arrest and apoptosis in *HL-60* cells	JNK is activated by KML001, resulting in reduced hTERT expression, and shortened TRF length and telomere, leading to G1 arrest and apoptosis	Yoon et al. (2015)
	Aged rats	Ischemic perfusion and increase plasma irisin levels	JNK inhibits HERT expression and exacerbates liver injury and aging	JNK inhibits HERT expression and exacerbates liver injury and aging	Bi et al. (2020)
	*MM-AN* cell line	T-oligo	JNK is activated, hTERT mRNA expression is declined, and shelterin complex proteins POT1/TRF2 is up-regulated	Activation of JNK signaling down-regulates hTERT expression, causing telomere dysfunction and DNA damage, leading to apoptosis	Chhabra et al. (2018)
	Human breast cancer cells and glioblastoma cells	Silver nanoparticles Ag-np	DNA-PKcs is activated, and JNK1 and NHEJ are up-regulated.	DNA-PKcs is activated and JNK1 is up-regulated to repair telomere damage and promote cell proliferation.	Lim et al. (2017)
Stem cells exhaustion	Mice adult hematopoietic stem cells (HESCs)	Knockdown the KMT2C and stimulation with IL-1	JNK and p38 are unable to be increased by IL-1 stimulation after KMT2C is knocked out and HESCs are unable to proliferate	JNK and p38 stimulation by IL-1 promotes excessive proliferation and exhaustion of HESCs, causing acute myelogenous leukemia and aging.	Chen et al. (2019)
	Human umbilical cord blood CD34(+) cell transplantation in immunodeficient mice	JNK-IN-8 and knockdown the c-Jun	Inhibition of JNK activity by JNK-IN-8, or targeting knockdown of c-Jun can preserve hemocyte differentiation multilineage	JNK activation promotes functional exhaustion and senescence of HESCs and progenitor cells (HSPCs) via c-Jun	Xiao et al. (2019)
	Human nucleus pulposus stem cells (NPSCs)	tert-butyl hydroperoxide	JNK-c-Jun is down-regulated by HSP70, restores mitochondrial potential, and guards the ultrastructure of NPSCs from exhaustion	JNK signaling up-regulates c-Jun and stimulates abnormal mitochondrial membrane potential, leading to ultrastructural disruption and exhaustion of NPSCs, resulting in disc degeneration and aging	Zhang et al. (2021)
	Drosophila	Jafrac1 (Thioredoxin peroxidase 1) and gene silencing	Low doses of ROS reduce differentiation defects and opa1RNAi promotes intestinal misdifferentiation	Defective mitochondrial fusion results in low ATP and high ROS, promoting JNK activation and affecting the misdifferentiation of iSCs	Deng et al. (2018)
	Human neural progenitor cells	iCRT-14	Inhibited JNK activation, ATF2 activation and differentiation of NSCs	Activates Wnt and β-catenin can promote the differentiation of NSCs via JNK/c-Jun/ATF2	Bengoa-Vergniory et al. (2014)
	Astrocytes	Focal cerebral ischemia/reperfusion	–	HMGB1 promotes JNK phosphorylation, facilitates the differentiation of NSCs, and repairs brain damage.	Li et al. (2014)
	Drosophila	–	–	Wnt activates JNK and promotes differentiation of ISCs by up-regulating the expression of Ccdn1, Axin2 and Lgr5 via c-Jun	Jiang H. et al. (2016)
	Male *Drosophila* germline-stem cells (GSCs)	–	–	Activation of JNK by TNF leads to changes in the *Drosophila* reproductive microenvironment and defective differentiation of GSCs	Chang et al. (2019)
Altered intercellular communication	Mice	Anisomycin	Activation of JNK reduces expression of Cx43	JNK inhibits Cx43 expression through c-Jun, which affects intercellular communication and leads to atrial fibrillation and aging	Yan et al. (2018)
	Rat *H9c2* cells	IS	IS elevates p-JNK levels and reduces Cx43 mRNA and expression	Activation of JNK leads to a decrease in Cx43, which affects intercellular communication and contributes to cardiovascular disease risk and aging	Changchihen et al. (2020)
	Human umbilical vein endothelial cells	AGEs, SP600125	AGEs promote JNK phosphorylation and increases Cx43, whereas inhibition of JNK reversed the increase in Cx43	JNK increases Cx43 in human umbilical vein endothelial cells	Zhao et al. (2019)
	hPDLCs	SP600125	Cx43 expression is down regulated	JNK activation inhibits Cx43 expression and promotes periodontal tissue atrophy and aging	Cao et al. (2023)
	hPDLSCs	SP600125	Connective tissue growth factor (CTGF) increases JNK activity, Cx43 expression is reduced, Panx1 is not reduced	JNK increases Cx43expression in hPDLSCs	Wu et al. (2022a)
	*SW1353* cells	Fumitremorgin C	Fumitremorgin C inhibits the phosphorylation of JNK, p38, which is reversed by SIRT1 inhibitors, which in turn promotes the production of MMPs	JNK promotes osteoarthritis formation and bone aging by facilitating the production of MMPs, causing COLA degradation	Zhou et al. (2022)
	Human dermal fibroblasts	Procyanidin B1	JNK phosphorylation is inhibited and MMP-1 production is reduced	Procyanidin B1 inhibits the phosphorylation of JNK, inhibits the production of MMP-1 and the degradation of collagen, and fights against skin aging	Cha and Kim (2015)
	Human dermal fibroblasts	TNF-α and GDHBA	TNF-α induction promotes to increase ROS, phosphorylation of c-Jun and NF-kB and COX-2, MMP-1 production, which is reversed by GDHBA	JNK is activated by TNF-α to produce MMP-1, which stimulates collagen degradation in aging skin	Kim K. S. et al. (2022)
	Hairless rats	Ultraviolet A (UVA) radiation	Leading to more ROS production, JNK phosphorylation and increased MMP-1	JNK activation by ROS promotes MMP-1 production and COLA degradation, altering intercellular communication and promoting skin aging	Lan et al. (2019)
	Human	300 muscle extension contractions	Monocyte chemotactic protein-1 (MCP-1) is abnormal, JNK is activated, and ECM is abnormal	JNK activation by MCP-1, resulting in ECM abnormalities as well as altered intercellular communication and senescence, may be implicated in the slow recovery of muscle-movement in old age	Sorensen et al. (2018)
Cellular senescence	CD8+ cells	–	TNF-α expression did not differ in T-cells of different ages, but there were differences in the degree of T-cell senescence	TRAF2 is differentially expressed during senescence and promotes T cell senescence by activating JNK	Gupta et al. (2018)
	Macrophage	Acrylamide (ACR)	ACR results in an increase in ROS, SA-β-gal, while knockdown of ATF-3 leads to a decrease in associated senescence markers	ATF3 activates p38 and JNK signaling by enhancing ROS/JNK production and promotes ATF3-dependent p53 expression, which in turn promotes p21 expression and cellular senescence.	
	HUVECs	SP600125 Gene silencing	IL-17A induction results in an increased proportion of G0/G1 phase cells, increased FTO production, increased SA-β-gal expression, and cellular senescence in HUVECs. FTO knockdown results in decreased levels of SA-β-gal, whereas SP600125 treatment results in decreased levels of FTO phosphorylation.	IL-17A promotes JNK phosphorylation, which in turn promotes FTO expression, an increase in SA-β-gal, an increase in the ratio of cells in the G0/G1 phase, senescence of HUVECs, and contributes to the aging of the vascular endothelium.	Li H. et al. (2023) and Li N. et al. (2023)
	*EA.hy926* cells and HUVECs	Dox, LPS and metformin	The increase in SA-β-gal expression and the levels of NF-κB and JNK phosphorylation are all reversed by the addition of metformin	JNK inhibition results in decreased SA-β-gal activity and reduces SASP, which inhibits endothelial cell senescence	Abdelgawad et al. (2023)
	Human nucleus pulposus cells	Kaempferol	Decreases levels of MMP3, COL1A and reduces phosphorylation of JNK, ERK1/2 delays senescence of NSCs	JNK promotes the production of MMP3, COL1A and the senescence of NSCs	Wang et al. (2023)
	Adipose tissue of senescent rats	l-Carnitine	Decreases JNK activity, SASP level and inflammatory factors level	l-Carnitine slows down adipocyte senescence and chronic inflammation in adipose tissue by inhibiting JNK activity to reduce SASP secretion as well as the release of inflammatory factors	Yang et al. (2019)
Circadian rhythm protein disorder	Osteoblast	Knockdown of BMAL1 gene	Increases JNK and ERK phosphorylation, enhances mTOR activity, decreases β-catenin expression and GSK-3 β phosphorylation	BMAL1 inhibits JNK activity, attenuates mTOR activity, enhances β-conjugated protein expression and GSK-3 phosphorylation, promotes osteoblast mineralization, and delays bone aging	Wei et al. (2023)
	Human VICs	Knockdown of BMAL1 gene	Activation of AKT, IκBα, p65, and JNK is decreased, and differentiation of VICs is reduced	BMAL1 inhibits p-JNK, and NF-κB/AKT/JNK is involved in VIC differentiation and promotes calcific aortic valve disease and aging	Jiang et al. (2023)
	Mice	Gene silencing	Increase in JNK phosphorylation and AP-1, thyroid cell proliferation	PER2 lack activates AP-1 through JNK signaling and promotes age-related thyroid hyperplasia disease and thyroid cancer.	Lee et al. (2022)
	Mice	SP600125	FGF21 is highly expressed more and BMAL1 is reduced	Neutrophil infiltration activates JNK, inhibits FGF21 production and promotes BMAL1 expression	Crespo et al. (2020)
	Mice	–	Increases in TNF-α, the level of JNK phosphorylation, and proliferation of intestinal epithelial cells	Deletion of BMAL1 leads to TNF-α production and JNK activation, resulting in excessive proliferation of intestinal epithelial cells and disruption of circadian rhythms	Stokes et al. (2017)
	Rat *C6* cells	SP600125	SP600125 reduces levels of CREB phosphorylation ultimately leads to a reduction in PER1 mRNA	JNK activates AP-1, which in turn promotes CREB phosphorylation and ultimately PER1 expression	Sugimoto et al. (2011)
Intestinal flora dysbiosis	Hippocampal neurons	Oxymatrine	Elevates levels of JNK phosphorylation and increases expression of Bax, Bcl-2 and caspase-3	LPS stimulation increases LDH, elevates phosphorylation levels of JNK and p38, increases the Bax/Bcl-2 ratio, promotes caspase-3 expression and hippocampal neuronal apoptosis, and causes neurological aging	Cai et al. (2017)
	*BV2* cells	Ace	Decreases levels of JNK phosphorylation and reduces COX-2 and IL-1β	Ace reduces intracellular inflammation, attenuates JNK phosphorylation, and down-regulates COX-2 and IL-1β expression	Ding et al. (2020)
	Drosophila	Clone	Overexpression of α-SYN in the gut, increased brain DUOX, ROS, and JNK phosphorylation	Overexpressed α-SYN travels to the brain via the cerebral-gut axis and accelerates AD progression and neurological aging via the DUOX-ROS-JNK pathway	Liu et al. (2022)
	Mice	HO-1	In response to TNF-α stimulation, the level of JNK phosphorylation is increased and TJs are destroyed, whereas HO-1 inhibits TNF-α-mediated TJs destruction	JNK is activated by TNF-α and destroys TJs via CASP3, disrupting intestinal barrier integrity	Zhou et al. (2021)
	Mice	DSS, mucin Akkermansia	ER stress markers EIF2α production, JNK activation, and reduction of TJs, *mucin Akkermansia* reduces TJs destruction	Activation of JNK by EIF2α is involved in the destruction of TJs and promotes the disruption of the intestinal barrier	Wade and Su (2021)
Pyroptosis	–	ASK-1	JNK-1 is activated, IL-1β, IL-18 and N-GSDMD are increased and thermoproteins are deposited	JNK-1 activates NLRP3, which promotes the production of IL-1β, IL-18 and N-GSDMD via CASP-1, and promotes thermoprotein deposition and cellular death, which ultimately results in plaque formation, causing atherosclerosis and vascular aging	Hoseini et al. (2018)
	Mice	H. capsulatum	Knockdown of Dectin, inhibition of cathepsin B release, inhibition of JNK activation, inhibition of IL-1β, NLRP3 and CASP1 production	Dectin-2 stimulates cathepsin B release, activates JNK, and promotes cellular pyroptosis caused by NLRP3, CASP1, and GSDMD, which in turn affects IL-1β production	Chang et al. (2017)
	Mice	miR-351	p-NF-κB, NLRP3, CASP1, N-GSDMD, JNK, MLK3 are significantly reduced	miR-351 directly regulates MLK3, which in turn activates JNK and promotes cardiac fibrosis through NLRP3 and CASP1	Wang et al. (2020a)
	*BV2* cells	LPS, SP600125	JNK phosphorylation is reduced, and both NLRP3 and CASP3 are declined	LPS-mediated JNK activation delays postoperative cognitive recovery in older adults through NLRP3 and CASP3 pathway-mediated glial cell pyroptosis	He et al. (2021)
	Mice *661 W* cells	eIF2α gene silencing	p-JNK/JNK ratio, increases ATF4, CHOP, and cleaves CASP-3	Activation of JNK by EIF2α stimulates ROS production, disrupts endoplasmic reticulum homeostasis and causes DNA damage, promotes the expression of CASP3 and the generation of N-GSDME, and leads to photoreceptor cell pyroptosis and retinal atrophy, which ultimately contributes to the aging of eyes	Topiwala et al. (2023)
Fibrosis	Human *Huh-7* cells	IL-1β, SP600125, Bay 11–7,082	IL-1β increases FGF21 and decreases β-Klotho levels. While SP600125 and Bay 11–7,082 eliminated IL-1β effects on FGF21 and β-Klotho	IL-1β stimulates the NF-κB/JNK pathway, increases FGF21 levels, and inhibits fibrosis in the liver	Lee et al. (2018)
	Mice	Transfect	FGF19 overexpression, increases BIM protein expression, inhibits JNK phosphorylation, inhibited TGF-β, and partial impairment of COLA, fibronectin, and α-SMA	Inhibition of JNK by FGF19 down-regulates TGF-β, which in turn partially inhibits COLA, fibronectin and α-SMA and suppressed lung fibrosis	Justet et al. (2022)
	*HL-1* cells	Hypoxia	Increases JNK phosphorylation, upregulation of c-Jun and ATF2, and increases expression of COL1A and COL3A, TGF-β1, and α-SMA	Activation of JNK stimulates c-Jun/ATF2 expression, which in turn promotes COLA and α-SMA expression, contributing to cardiac fibrosis and aging	Tsai et al. (2021)
	Dusp1 transgenic mice	Dusp1	Dusp1 reduces JNK phosphorylation, MFF production, and mitochondrial fission	Activation of JNK promotes MFF production, stimulates mitochondrial division, promotes cytC release, energy disruption, mitochondrial potential reduction, and promotes fibrosis in the kidney	Sheng et al. (2019)

## 10 Target JNK signaling for aging and its associated diseases

As mentioned previously, JNK signaling is implicated in various aging processes and contributes to the development of age-related diseases. Notably, the activity of JNK progressively increases with age (Rutkute and Nikolova-Karakashian, 2007). Consequently, targeting JNK signaling presents a promising strategy for anti-aging interventions, with JNK inhibitors emerging as potential therapeutic agents. Over the past decade, numerous small molecule and peptide JNK inhibitors, based on diverse molecular scaffolds, have been discovered, although only a subset has advanced to clinical trials (Ulgherait et al., 2021). These JNK inhibitors hold the potential to delay aging and treat age-associated conditions (Table 2). Notable examples of JNK inhibitors include SP600125, CC-930, CC-401, CC-90001, and CEP-1347 (Han, 2008; Zhdankina et al., 2023).

**Table 2:** Targeting JNK for aging and related diseases.

Compounds	Model	Dose	Time	Effect	References
SP600125	Rotifera	1 μM	4 days	Average life expectancy increases by 35%, maximum life expectancy increases by 37%	Snell et al. (2014)
	APPswe/PS1dE9 mice	30 mg/kg/d	12 weeks	Fewer Aβ proteins are produced and synaptic loss is attenuated to alleviate progressive cognitive dysfunction with age	Zhou et al. (2015)
	C57BL/6 mice	4 mg/kg/d	7 or 14 days	Suppress age-associated reduction in 17β-estradiol and progesterone levels and premature ovarian failure	Park et al. (2018)
	Male Lewis rats	30 mg/kg/d	14 days	Moderately reduce paw swelling in arthritic mice	Han et al. (2001)
	BALB/c mice	30 mg/kg/d	24 days	Significantly inhibit breast cancer tumor growth	Li et al. (2020)
	Mice	50 mg/kg 5 times/week	Till mice died	Significantly reduce pancreatic cancer growth and promote survival in mice	Takahashi et al. (2013)
	Human lung adenocarcinoma *A549* cells and human H446 SCLC cells	0–4 μg/mL	1 h	Inhibit the expression of lung cancer resistance genes at the mRNA and protein levels and promote chemosensitivity and apoptosis in lung cancer cells	Xu et al. (2017)
Sotetsuflavone(SF)	Crohn’s disease (CD) mice	50 μM	12 h	SF inhibits inflammatory response and intestinal damage.	Jia et al. (2023)
CC-930	Mice	25, 50, 100 and 150 mg/kg Twice/day	14 days	Pulmonary fibrosis is reduced by 18–32%.	Krenitsky et al. (2012)
	Rats and mice	60 mg/kg Twice/day	–	Significantly reduce renal failure.	Grynberg et al. (2021)
	Human	75 mg/d or 200 mg/d	6 days	–	van der Velden et al. (2016)
	Human patients with pulmonary fibrosis	50 mg/d, 200 mg/d	4 weeks	Patients on the low dose are more stable, while some patients on the high dose develop elevated liver transaminases and cardiac abnormalities, which are more serious and have been discontinued from the study	
CC-90001	Health human	Single:10–720 mg/d Multiple: 30–480 mg/d	7 days	Common adverse reactions are mild or moderate nausea or dizziness. But overall, it’s safe	Ye Y. et al. (2022)
	Human patients with pulmonary fibrosis (clinical phase 1b trial)	200 mg/d or 400 mg/d	12 weeks	The absence of a significant decrease in lung capacity relative to baseline is usually safe and well tolerated	
	Human patients with pulmonary fibrosis (clinical phase 2 trial)	200 mg/d or 400 mg/d	Screening period: 4 weeks Treatment period: 24 weeks Active-treatment extension: 80 weeks	–	Popmihajlov et al. (2022)
CEP-1347	Parkinson’s disease patients	50 mg/d	4 weeks	Slight nausea, dizziness. Safe and well tolerated	Parkinson Study Group (2004)
	Early Parkinson’s disease patients	10/25/50 mg Twice a day	4 weeks	Treatment is ineffective in the early stages of Parkinson’s	Parkinson Study Group PRECEPT Investigators (2007)

Currently, research on JNK targeting primarily focuses on the following areas: Firstly, inhibiting JNK activity and interfering with JNK expression have demonstrated lifespan extension and deceleration of aging. For instance, the average lifespan of rotifers was extended by 35% at a concentration of 1 μM of the JNK inhibitor SP600125, and RNAi knockdown of the JNK gene increased the average lifespan of rotifers by 51% (Snell et al., 2014). Moreover, the use of SP600125 delay aging-related symptoms, including cognitive decline in an AD mouse model (Zhou et al., 2015). Additionally, SP600125 inhibited JNK-mediated age-related ovarian failure in mice (Park et al., 2018). Furthermore, in osteoblasts, SP600125 prevented JNK-induced apoptosis, thereby offering potential protection against osteoporosis (Gunaratnam et al., 2013). Secondly, targeting JNK also exhibits anti-inflammatory effects, as inhibiting JNK activity can attenuate the inflammatory response and hold therapeutic potential for various inflammatory conditions (Bennett et al., 2001). In models of inflammatory diseases such as rheumatoid arthritis and Crohn’s disease, the use of JNK inhibitors significantly reduces the inflammatory response (Han et al., 2001; Xu et al., 2019; Jia et al., 2023).

Thirdly, targeting JNK holds potential for neuroprotection (Braithwaite et al., 2010). As the aging process unfolds, the nervous system undergoes gradual degeneration due to nerve cell apoptosis. JNK is involved in the mechanisms of neuronal death and apoptosis (Okuno et al., 2004). Inhibiting JNK activity can protect neuronal cells from damage, thereby offering therapeutic benefits in various neurodegenerative diseases (Kumar et al., 2015). For instance, in a mouse model of Parkinson’s disease (PD), the use of JNK inhibitors significantly attenuated nerve damage and improved motor function (Wang et al., 2009). Moreover, targeting JNK can also exert an anti-tumor effect. JNK plays a crucial role in the growth, division, and metastasis of tumor cells, and cancer is a class of diseases closely associated with aging (Lee, 2020; Sedrak and Cohen, 2023). By utilizing JNK inhibitors like SP600125 to suppress JNK activity, the growth and dissemination of tumor cells in cancer models such as breast, pancreatic, and lung cancer can be inhibited, thereby demonstrating preventive and therapeutic effects against a variety of cancers (Takahashi et al., 2013; Joo et al., 2017; Xu et al., 2017; Li et al., 2020).

Currently, JNK inhibitors can be categorized into ATP-competitive inhibitors, allosteric inhibitors, bidentate-binding inhibitors, and dual inhibitors (Ulgherait et al., 2021). ATP-competitive inhibitors of JNK comprise a group of small molecule compounds; however, their efficacy is diminished at high ATP concentrations (Graczyk, 2013). Among the extensively studied JNK inhibitors are SP600125 and AS601245, which exhibit good inhibitory effects on multiple JNK isoforms (Wu et al., 2020). Research has demonstrated the utility of SP600125 in cellular and animal models of inflammation, cardiovascular aging, brain degenerative diseases, and certain cancers (Sharma et al., 2010; Mili et al., 2016; Wu et al., 2017). Nonetheless, SP600125 lacks selectivity for JNK isoforms, limiting its application in recent developments (Wu et al., 2020). To address the issue of ATP competition in JNK inhibitors, stronger selectivity is an effective solution to mitigate potential reversal, especially in cells with high ATP concentrations resulting from robust metabolism.

In comparison to ATP-competitive inhibitors, JNK variant inhibitors offer greater selectivity due to the variability of their target sites. For instance, pepJIP1, D-JNKI-1, and JIP10-∆-TAT exhibit improved selectivity for JNK isoforms, albeit with reduced activity and selectivity (Borsello et al., 2003; Kaoud et al., 2011; Stebbins et al., 2011). Bidentate-binding inhibitors are JNK inhibitors that simultaneously target both ATP and JNK inhibitor peptide (JIP) binding sites (Ulgherait et al., 2021). By combining appropriate bifurcated molecules at these binding sites, highly selective JNK inhibitors can be designed. The existing JNK inhibitor, SR9444, demonstrates reasonable selectivity for JNK1/2; however, its cell penetration and cellular activity are suboptimal (Feng et al., 2013). Dual inhibitors are a type of JNK inhibitor that independently bind to both the JIP and ATP binding sites, offering enhanced cellular activity and stability (Chen et al., 2009). These compounds exhibit selectivity for JNK1/2 over JNK3 (De et al., 2011). Nevertheless, their specificity requires further improvement, and they have not yet been employed in clinical trials related to aging.

Several JNK inhibitors targeting aging-related conditions have already progressed to clinical stages. CEP-1347, a derivative of K-252α derived from the Narcodiopsis bacterium, promotes neuronal cell survival by inhibiting JNK activation (Kaneko et al., 1997). It is currently undergoing phase clinical trials for Parkinson’s disease treatment, although its efficacy in early-stage Parkinson’s treatment is limited (Parkinson Study Group PRECEPT Investigators, 2007). CEP-1347 has also reached phase III clinical trials for JNK-associated age-induced heart-related disorders (Liu et al., 2018). Another JNK inhibitor, CC-930, exhibits specificity for JNK1 and is currently in phase II clinical trials for idiopathic pulmonary fibrosis, but it presents some cardiac adverse effects (Krenitsky et al., 2012). Similarly, CC-90001, an oral JNK inhibitor, is in phase II clinical trials for idiopathic pulmonary fibrosis (Popmihajlov et al., 2022). AGI-1067, a JNK inhibitor with anti-inflammatory properties, has been applied in phase III clinical trials for atherosclerosis and myocardial infarction (Tardif et al., 2003).

JNK is a crucial signaling pathway that plays a significant role in normal physiological processes (Jamal et al., 2023). Consequently, the use of JNK inhibitors may have side effects on normal physiological functions. For instance, JNK inhibitors might interfere with cellular stress responses, leading to reduced immune function or increased susceptibility to infections. Furthermore, long-term usage of JNK inhibitors may result in potential toxic effects. Studies have indicated that prolonged use of JNK inhibitors can cause liver damage, kidney toxicity, and cardiovascular issues (van der Velden et al., 2016). Additionally, JNK inhibitors may disrupt cell cycle regulation, leading to abnormal cell proliferation or mutations. For example, the activation of upstream genes such as MKK4 and MKK7 can be enhanced or attenuated by JNK inhibitors (Lombard, 2019). Moreover, the use of JNK inhibitors can interact with other drugs, potentially increasing their side effects or decreasing their efficacy. Therefore, careful consideration of drug interactions is necessary when utilizing JNK inhibitors.

JNK inhibitors have been extensively investigated as potential treatments for age-related diseases. However, there are several factors that need to be considered when evaluating their safety. Currently, JNK inhibitors are available in the form of small molecules and peptides, with small molecules having better pharmacokinetic properties. However, small molecule JNK inhibitors often exhibit poorer specificity, leading to off-target effects and increased cytotoxicity, which can exacerbate aging and associated diseases (Ulgherait et al., 2021). Dual inhibitors face similar challenges, as they may have greater potential for efficacy but suffer from reduced subtype specificity. This limitation significantly hampers the clinical use of JNK inhibitors for anti-aging purposes and poses a major obstacle to their development. Additionally, JNK inhibitors still have drawbacks such as poor cell permeability, instability, and short *in vivo* half-life. These limitations can potentially be addressed through the use of nanomedicine-based targeted delivery systems to improve drug transport to the affected tissues. Efforts should focus on developing JNK inhibitors with high efficiency, strong specificity, and enhanced stability.

In summary, JNK inhibitors hold tremendous research potential as potential drugs for the treatment of age-related diseases; however, safety profile requires further investigation and validation. More clinical trials and toxicity studies are necessary to ensure their safety and efficacy before clinical application. Furthermore, since research on targeting JNK is still in its early stages, further studies are needed to elucidate its mechanism of action in aging and age-related diseases, as well as to discover more effective drug candidates. Given the multifaceted nature of aging, the most effective strategy to extend healthy lifespan may involve a combination of therapies that simultaneously target multiple pathways. Therefore, investigating multi-targeted anti-aging combinations may represent a promising research direction for the future (Snell et al., 2014).

## 11 Current challenges

The JNK signaling pathway plays a significant role in the aging process, and a limited number of JNK inhibitors have been tested in clinical trials. However, our understanding of the precise regulatory pathways and downstream factors associated with JNK in aging remains incomplete. Aging is a multifaceted physiological process, and the involvement of JNK in the aging signaling pathway and its potential interventions face several shortcomings and challenges.

First and foremost, the JNK signaling pathway exhibits a dual nature during the aging process. In clinical and animal models, inhibiting JNK activity promises in delaying aging and has therapeutic potential for aging-related diseases. Conversely, genetic deletion or chemical inhibition of JNK improves disease status in animal models (Ulgherait et al., 2021). However, it is important to note that these effects can be either positive or negative. For instance, when JNK is activated in the brain, it leads to a prolonged lifespan, whereas moderate activation of JNK in midgut intestinal stem cells (ISCs) and enteroblasts significantly shortens lifespan (Gan et al., 2021). Additionally, the role of JNK in age-related cancers is context-dependent, varying based on different circumstances (Wu et al., 2019). Secondly, there is still much work to be done in order to clearly and effectively target the JNK pathway in clinical settings. The use of JNK inhibitors alone may not be feasible due to interspecies differences and tissue specificity. Therefore, the primary focus should be on identifying the causal role of activated JNK in the manifestations of aging. This would enable the classification of different types of aging and the identification of causative agents for aging-associated diseases that are dependent on activated JNK signaling.

Furthermore, to overcome the non-specificity of JNK targeting, it is crucial to identify specific substrates or JNK isoforms that are relevant to a particular cellular state or cell type (Sabapathy, 2012). It is worth noting that the current focus of drug development is primarily on inhibiting JNK activity, while drugs that activate JNK may have potential benefits for specific aging processes and aging-related diseases. Additionally, some existing studies on aging mechanisms have not thoroughly explored the role of JNK isoforms. Moreover, while the JNK signaling pathway is considered a key regulatory pathway in aging, studying this pathway alone may not comprehensively address the initiation and progression of aging. The aging process and aging-related diseases are often influenced by multiple genes, necessitating the consideration of other signaling pathways and molecular mechanisms in an integrated manner to better understand and intervene in the aging process. A comprehensive study of aging as a holistic process could aim to connect different aspects of aging, such as how JNK is involved in circadian rhythm disorders caused by sleep deprivation, aging induced by dysbiosis of intestinal flora, inflammation, and neurodegenerative diseases. This approach would provide a more comprehensive understanding of the role of JNK inhibitors in aging. By connecting these various aspects, research can progress from individual points to a more extensive and interconnected framework. Lastly, there is currently a lack of studies on the involvement of JNK in circadian rhythm disorders and intestinal flora dysbiosis, particularly in relation to aging. Further research in these areas would provide a deeper and more comprehensive understanding of the senescence pathway involving JNK. This knowledge would also help overcome the barriers that hinder the clinical application of JNK-targeting drugs, leading to improved selection and clinical implementation.

In the future, it is crucial to further investigate the interplay between JNK isoforms and other signaling pathways to gain a comprehensive understanding of the complex nature of the aging process. It is essential to uncover the specific mechanisms by which JNK influences cellular senescence and organismal aging, with a particular emphasis on highlighting the divergent roles and differences of JNK in various cellular senescence and aging mechanisms. Furthermore, it is important to enhance the collection and analysis of human research data related to the JNK pathway in clinical studies. Conducting large-scale population studies will enable a deeper exploration of the relationship between the JNK pathway and human aging, providing a scientific foundation for personalized interventions. Developing intervention strategies targeting the JNK pathway is also crucial. The effects of modulating JNK activity or altering the expression of JNK-related molecules on the aging process should be thoroughly investigated, with the aim of identifying potential therapeutic targets and drugs. Additionally, exploring upstream signaling events that indirectly regulate JNK activity is essential. Moreover, it is worth exploring different JNK-targeting compounds derived from natural plant sources and considering the use of drug combinations to overcome limitations. Additionally, the potential of utilizing promising targeting nanocarriers, such as ferritins and GP96, for JNK drug delivery should be tested. Although there are still gaps in our understanding of the JNK signaling pathway during aging, overcoming these challenges is expected to lead to significant breakthroughs in the prevention and treatment of aging-related diseases.

## 12 Concluding remarks and perspectives

**Figure 8:**
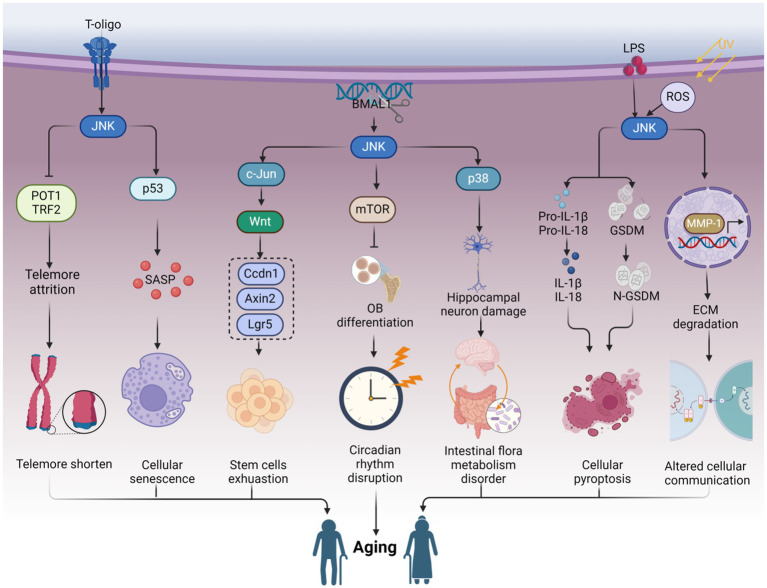
JNK signaling regulates aging through multiple pathways.

The JNK signaling pathway plays a significant role in the aging process. Activation of JNK signaling triggers the release of inflammatory factors from cellular pyroptosis, leading to alterations in cellular communication and fibrosis. JNK activation induces macrophage senescence and promotes senescence and programmed cell death in neural progenitor cells (NPCs). In *Drosophila*, JNK activation promotes the progression of Parkinson’s disease through disruption of the brain-gut axis and intestinal barrier. Additionally, in the absence of BMAL1, p-JNK levels increase in osteoblasts, resulting in impaired osteoclast differentiation and mineralization, which contributes to osteoporosis. Moreover, elevated JNK activity in hairless mice further accelerates skin aging. Notably, dysregulation of the circadian rhythm protein PER2 activates the AP-1 transcription factor via the JNK pathway, contributing to age-related thyroid hyperplasia. Furthermore, JNK activation inhibits the transcriptional activity of the Cx43 gene promoter, leading to decreased Cx43 expression and impaired intercellular communication, which can contribute to atrial fibrillation and cardiovascular disease. Activation of JNK reduces hTERT activity and disrupts Shelterin structure, resulting in telomere shortening in leukemia KG1 cells, as well as inducing G1 phase arrest and apoptosis in *HL-60* cells. However, under the influence of elevated plasma irisin levels, JNK activation increases stem cell telomerase activity and lengthens telomeres. Activation of JNK signaling promotes the activation of the NLRP3 pathway, leading to GSDMD-mediated cellular pyroptosis, further contributing to the development of atherosclerosis, systemic sclerosis, and myocardial fibrosis. Finally, several JNK-targeting drugs, including CEP-1347, CC-930, CC-90001, and AGI-1067, have already reached the clinical stage. Additionally, natural compounds such as *nypa fruticans* extract and quercetin have shown promising results in animal models and isolated experiments (Figure 8).

Currently, the main limitation of JNK intervention in anti-aging drugs lies in the fact that JNK is a central hub in multiple signaling pathways. Inhibiting JNK alone may lead to various adverse reactions within cells. While positive results have been observed in *in vitro* experiments, clinical trials have shown some side effects, including hepatotoxicity and cardiac discomfort. Therefore, the concentration and dosage of JNK intervention drugs that can achieve the desired anti-aging effect, as well as the need for adjuvants, require extensive experimentation for verification. To fully utilize JNK intervention drugs, it is important to achieve a dual role in promoting senescence-related pathways and inhibiting anti-aging pathways. For example, these drugs could promote apoptosis in cancer cells and damage the telomeres of cancer cells, while slowing down senescence in normal cells. Additionally, by considering upstream factors of the JNK senescence-related pathway, specific JNK isoforms, applicable cell types, and other senescence-related pathways, selecting the appropriate JNK intervention drug targeting therapy may yield favorable anti-aging effects. Considering the adverse effects observed in humans, it is advisable to conduct drug testing on mammals and primates closely related to humans. Successful drug testing should be followed by the addition of more clinical data to fill gaps and provide a foundation for further research.

In the future, based on mature research on JNK signaling-related mechanisms and the clinical application of JNK intervention drugs, the development of orally-administered drugs that can reach all parts of the body through the brain-gut axis and the circulatory system is a possibility. However, this is a more challenging task compared to targeted JNK therapy. It is worth noting that while stem cell failure is indeed a sign of aging, an excess of stem cells in the human body may lead to cellular accumulation, increased intercellular competition, reduced self-renewal and differentiation capacity, and impaired tissue and organ function. Excessive stem cells may also increase the risk of cellular mutations and disease. Therefore, it is important to explore how JNK intervention drugs can accurately regulate the number of stem cells and their differentiation direction to maximize their anti-aging efficacy while minimizing potential risks. Furthermore, considering the relationship between intestinal dysbiosis and aging, research on fecal microbiota transplantation or identifying beneficial microbiota abundances in healthy long-lived individuals to develop agents for indirect intervention in the aging process and related diseases involving JNK is also a promising area of future investigation. We believe that in the future, mature JNK clinical intervention drugs will likely be a gradual shift into healthcare products, or the development of anti-aging drugs tailored to different age stages and treatments for various types of aging-related diseases. This will enable humans to live longer, healthier lives, free from disease, and enjoy a high quality of life.
